# Assessment of ecofriendly carbon capture using *Bacillus subtilis* induced calcium carbonate precipitation with focus on applications mechanisms and cost efficiency

**DOI:** 10.1038/s41598-025-06688-1

**Published:** 2025-07-01

**Authors:** Amal W. Danial, Raghad M. M. Hasan, Ghada Abd-Elmonsef Mahmoud, Refat Abdel-Basset

**Affiliations:** Botany and Microbiology Department, Faculty of Science, Assiut University, Assiut, 71516 Egypt

**Keywords:** *Bacillus subtilis*, BICCP, Calcium carbonate, Urease, Nitrogenase, Carbon capture, Water holding capacity, Mineral sorption, Crack healing, Biological techniques, Biotechnology, Ecology, Evolution, Microbiology, Physiology, Climate sciences, Environmental sciences

## Abstract

This work focuses on exploiting the naturally occurring microbial calcium carbonate precipitation catalyzed by microbial consortia within lakes and oceans biogeochemistry for carbon dioxide removal from atmosphere. In this work, *Bacillus subtilis* OQ119616 was used for carbon dioxide sequestration in equi-molar concentrations into *Bacillus*-induced calcium carbonate precipitation (BICCP). As this process requires alkaline media, urea degradation by urease and nitrogen fixation were traced. BICCP has been formed from calcium salts in the following order: chloride > nitrate > acetate > citrate. However, conversion efficiency percentage (CE%) of calcium salts to CaCO_3_ exhibited a different attitude of citrate > acetate > chloride > nitrate. Calcium citrate is excluded from consideration. Acetate, however, is the most efficient salt; it significantly exhibited the highest CE%, with the least cost and highest economic feasibility. The wide range in quantities, efficiency and feasibility indicates the importance of the salt anion in BICCP. In addition, BICCP exhibited applicability in healing concrete cracks, improving field capacity of sand soil and the subsequently improved seed germination of *Vicia faba*. BICCP was also accompanied by adsorption of heavy metals as partial purging of waste/sewage water for hygiene/reuse. *Bacillus subtilis* exhibited the ability to perform MICP, utilizing various calcium salts in the following order: chloride > acetate > nitrate > citrate. However, acetate is the most efficient salt of calcium to be converted to calcium carbonate precipitate by *B. subtilis*, as it exhibited the highest conversion efficiency percentage (g/g %), with the least cost and highest economic feasibility. Carbon dioxide removal (CDR) occurs at simultaneous equity to CaCO_3_ precipitation at mole/mole ratios. Economic feasibility (US$/m^3^) showed that BICCP may be applicable in CDR for cleansing carbon dioxide inside closed systems and for environmental safety. The bacterially induced CaCO_3_ proved successful applicability in improving the field capacity of sand soil and growth of *V. faba*, healing concrete cracks and sorption of heavy metals for depolluting sewage/wastewater for hygiene reuse. BICCP could repair concrete cracks of 1–2 mm wide in 7 days by 210 * 10^6^ cells/mL. Adsorption of heavy metals (Pd, Zn, Cd and Cu) for partial removal of contaminants in/from waste/sewage water for hygiene reuse.

**Supplementary Information:**

The online version contains supplementary material available at 10.1038/s41598-025-06688-1.

## Introduction

Due to its high and substantial applicability potential, calcium carbonate particles are under considerable investigation for developing novel synthesis methods. Naturally, calcium carbonate can be formed at very low solubility levels in pure water before precipitating. Its solubility in pure water is as low as 13 mg L^−1^ at 25 °C^1^, increases relatively with decreasing temperature and increases in rainwater saturated with carbon dioxide, due to the formation of more soluble calcium bicarbonate. However, the microbially-induced calcium carbonate precipitation (MICP) is specifically important as it is much more in quantity and stability, continuous, of special importance and applicability and furthermore, it is considered green according to Preksha et al.^2^. MICP depicts an exogenous or endogenous microbial activity during heterotrophic growth of fungi (e.g.^3^) and bacteria^4^ or during photoautotrophic growth of cyanobacteria^5^. MICP occurs in natural environments of microbiota including water, soils, tufas and biofilms. The presence of MICP-forming microorganisms in eutrophic lakes indicates their significance in biomineralization at biogeochemical structures. The microbially induced calcium carbonate precipitation is a widespread phenomenon in the biological world^6,7^. Not only viable cells but also their dead bodies or their mucilaginous sheath (capsular polysaccharides of bacteria or the exopolysaccharides of cyanobacteria) as well as fungal chitin would act as nucleation sites for CaCO_3_ crystallization, by binding Ca^2+^ onto their carboxylic groups. Furthermore, the microbial strain performing MICP (eg,^8^) as well as the type of calcium salt provided^9^ determines the morphology of MICP crystals. In this respect, CaCO_3_ exists in three types of polymorphs: vaterite, aragonite, and calcite with varying shapes such as needle, spherical, and rhombohedral, respectively^8^. Aragonite is the metastable form of CaCO_3_ with an orthorhombic crystal system, which is an important constituent of pearl secreted by mollusks and other related invertebrates^10^.

The metabolism of MICP requires sufficient Ca^2+^, an alkaline pH and a suitable microorganism(s). The latter plays dual roles; their cell walls act mechanically as nucleation sites^11^, in addition to their metabolic processes such as photosynthesis, respiration, sulfate, nitrate or sulfide reduction and ureolytic activity, as driving mechanisms for MICP (e.g.^12^). Although these metabolic pathways are diverse, either of them drives MICP by saving CO_2_, as a component of calcium carbonate and ammonia, to shift the pH around the cells into alkalinity; both are prerequisites for the MICP process. Prominently, ureolytic activity of urease enzyme has been found as the major mechanism catalyzing MICP via CO_2_ and ammonia production during urea hydrolysis (e.g.^4^). In this respect, Castro-Alonso et al.^13^ reported on a series of complex reactions of urease, calcium, and carbonate during MICP surrounding the cells. Besides being a component of CaCO_3_, Ca^2+^ is inductive to urease activity resulting in a pronounced upregulation of MICP^8^: Subsequent to the ubiquitous coprecipitation of calcium and carbonate, chemically and/or microbially to form calcium carbonate, the bioavailability of calcium becomes limited in natural water bodies^14^, down to a threshold considered critical for the survival of many Ca^2+^-dependent organisms e.g. *Daphnia*^15^, which, in turn, acts as rate-limiting to growth and eutrophication of aquatic microbiota.

Calcium carbonates, in micrometer or nanometer dimensions, are considered chemically inert and have numerous applications such as biosensing, drug delivery, and filler material in plastic, paper, paint, sealant, and adhesive industries. The unusual properties of calcium carbonate-based nanomaterials, such as biocompatibility, high surface-to-volume ratio, robust nature, easy synthesis, and surface functionalization, and ability to exist in a variety of morphologies and polymorphs, make them an ideal candidate for both industrial and biomedical applications^1^. Of the paramount importance of MICP is carbon capture and sequestration, as carbon is coprecipitated simultaneously with calcium to form calcium carbonate precipitations, which is usually stored in durable geological, terrestrial structures such as soil catchments of water bodies. Therefore, MICP is a natural phenomenon promising to be exploited intentionally on the purpose of carbon dioxide removal (CDR) from that already emitted into the atmosphere for a net zero future or otherwise in industrial products.

MICP is formed naturally by the aid of microbial consortia over long periods of time. However, the hypothesis of this work is to explore whether a uni*-*bacterial culture is able to perform MICP up to applicable quantities and approaches within their life span. Accordingly, the ability and magnitude of a local, newly isolated strain of *Bacillus subtilis* to perform the process of bacterially induced calcium carbonate precipitation (BICCP) was explored in this work. The dependence of BICCP magnitude as well as its morphs on Ca^2+^ salt type was studied at different concentrations of calcium citrate, acetate, nitrate, or chloride. As MICP requires alkaline media, ammonification substantially, urea degradation by urease and nitrogen fixation by nitrogenase were subsequently traced to elucidate their role in saving the required ammonia and to detect the effect of the applied treatments on their activities.

The applicability of the produced BICCP was pursued in several aspects. Specifically, utilization of BICCP as a sink for CO_2_ capture was postulated and hypothetically calculated as Kg calcium carbonate formed per Kg of calcium salt given to the bacteria and as Kg fixed CO_2_ per Kg of calcium carbonate (or as mole/mole). Costs and economic feasibility of calcium salts and CO_2_ conversion into CaCO_3_ were also considered. Additional uses of BICCP have also been pursued; these are namely, heavy metals adsorption to decrease pollution of waste sewage effluent for hygiene environment or reuse, improving field capacity of sand soil for upgrading its agricultural validity for germination and growth of *Vicia faba* seeds as well as repairing concrete cracks.

## Materials and methods

### Isolation of BICCB bacteria

Bacteria were isolated from soil samples collected from different localities in Sahel Salim, Assiut governorate. Samples inoculated in nutrient agar medium to isolate and purify heterotrophic bacteria for 1 day incubation period at 30 °C.

### Bacterial identification

Morphological and biochemical tests for Gram stain, gelatin hydrolysis, nitrate reduction, oxidation–fermentation of glucose, oxidase test, catalase, indole and urease production were performed as described by Barrow and Feltham^16^, motility, and fermentation of glucose, mannitol, sucrose. The isolated bacteria were classified using methods mentioned by Hussein et al.^17^. Diagnostic media of general use in identification were prepared as recommended by Harrigan and Mecance^18^ and specific media for the identification of isolates were also prepared. Furthermore, genotypic identification of the isolates was carried out on the bases of 16S rRNA base sequence analysis after extraction of total genomic DNA^19^.

### Molecular identification

The primer used was “F (5′-AGA GTT TGA TCC TGG CTCAG-3′)” with a GC clamp and “R (5′-GGT TAC CTT GTT ACG ACT T-3′)” at the annealing temperature of 65 °C were used for the PCR amplification of the variable region of 16S ribosomal DNA (rDNA) from the purified genomic DNA. The PCR product is made by a Korean company by ABI 3730xl DNA sequencer. Sequence analysis was conducted based on the online databases using BLAST by MEGA 3.1 software for phylogenetic analysis. The neighbor- joining method was used to build the phylogenetic tree.

### Nucleotide sequence accession numbers

The nucleotide sequence of the bacterial isolate (*B. subtilis*) was deposited in the database of GenBank nucleotide sequence under accession number of OQ119616 and the Phylogenetic tree including the strains was illustrated.

#### Organism and inoculation

The isolated strain was grown in 250 ml Erlenmeyer flasks containing 100 ml minimal medium in a shaker incubator for 3 days until reaching maximum turbidity (approximately 1 × 10^9^ cells ml^−1^). Bacterial inoculum population was estimated with plate dilution method and total count^20^. Ten ml of the log phase bacterial culture was inoculated into each pot, watered regularly to maintain the soil at field capacity.

### Experimental set up

Cultures of the locally isolated strain, which has been identified as *B. subtilis* OQ119616, were incubated at different Ca^2+^ concentrations of different salts (citrate “Cit”, acetate “Ac”, chloride “Cl”, or nitrate “N”) supplemented into calcium-free minimal medium (control culture). Concentrations of calcium salts were dependent on the specific solubility coefficient of each salt as follows: citrate 0.23, 0.46, 0.69 and 0.96 g; acetate 0.5, 1.0, 1.5 and 2.0 g; chloride 5, 10, 15 and 20 g, and nitrate 5.0, 10, 15 and 20 g). Culture media were inoculated with 10 ml of 5 days old cells in conical flasks capped and shaken for 4 weeks at 28 °C ± 2.

### Growth

Growth of the variously treated cultures was followed as optical density (O.D._660 nm_) over a period of 5 days. The culture started at 0-time with OD_660_ nm of 0.2A accounting to 21 * 10^7^ cells/mL.

### Ammonia content and urease activity

Urease enzyme (UE) activity was assayed spectrophotometrically as ammonia following the procedure of Brink^21^. Use Stuart’s Urea Broth (yeast extract 0.1 g potassium phosphate monobasic 9.1 g potassium phosphate, dibasic 9.5 g, urea 20 g, phenol red 0.01 g) in 1L. To prepare the urea base, dissolve the first four ingredients in 900 ml of distilled water, and autoclave at 121 °C and 15 psi for 15 min and filter sterilize (0.45-mm pore size) in 100 mL of distilled water then take 1 mL of subculture of bacteria and different amounts of calcium salt, and measure color at 450 nm daily for 5doys.

Ammonia accumulated in the different culture media at the end of the experimental period (30 days) was assessed as mentioned above in urease-liberated ammonia.

Urease activity of control and calcium-treated cultures was assessed daily over the growth period (5 days). The enzyme activity was assessed as ammonia following the procedure recommended as above.

### Nitrogenase activity

Nitrogenase activity of control and calcium-treated cultures was assessed daily at 24 h intervals throughout their growth cycle over the growth period (5 days). Nitrogenase activity, as acetylene reduction (ARA) following Hardy and Knight^22^ and Hardy et al.^23^. The bacterial cells were placed in 500 ml bottles sealed with a rubber septum; 50 ml of air were taken, and the same volume of acetylene gas was introduced into the bottle, incubated at 37 °C. Then, samples from the bacterial atmosphere in bottles were withdrawn and injected into the gas chromatograph. A calibration curve was constructed using pure ethylene. The nitrogenase activity or (ARA) is expressed directly as µM C_2_H_4_ produced per mg bacterial protein.

### Bacterially induced calcium carbonate precipitation (BICCP)

In conical flasks, volumes (200 mL each) of minimal media containing peptone, beef extract (5 g/L each) in addition to 20 g/L urea and one of four calcium salts (chloride, citrate, acetate and nitrate) at four concentrations of each. Cultures were inoculated with equal aliquots of the bacterium *B. subtilis*, incubated at 25 °C under aerobic conditions for 30 days then precipitates of the bacterially-induced calcium carbonate (BICCP) were collected by filtration, dried in an oven at 100 °C for 24 h and their dry weight (x g/200 mL) was assessed and validated to Y g/L.

Observations and morphological studies of the bacterial precipitates obtained were further carried out by electron microscopy and XRD.

- Scanning electron microscopy (JSM 5400 LV) equipped with qualitative and quantitative facility, Energy dispersive—X ray microanalysis (EDX) and wave length dispersive—X ray microanalysis (WDX) for both investigation and examination of topographical features and fracture surface details of different specimens and elemental analysis of different samples. Shapes of calcium carbonate produced by *B. subtilis* as influenced by the provided calcium salts are presented.
- Transmission electron microscopy (JEM 100 CXII) for investigation and examination of micro structural details of different specimens.
- The X-ray diffraction (XRD) analysis XRD of CaCO_3_ was performed using X-ray diffractometer (Model PW 1710 control unit Philips Anode Material Cu, 40 kV, 30 M.A, optics: Automatic divergence slit) with Cu Kα radiation λ = 1.5405 Å over a wide range of Bragg angles (20° ≤ 2θ ≤ 80°). Elemental analysis of the sample was examined by energy dispersive analyses of X-rays with JED- 2300 instrument.

### The pH of the culture media

After filtration of all cultures and the pH values of the supernatants were measured with pH meter (HI 12216, HANNA, Romania).

### Cells viability

Viability of bacterial cells was assessed by sub-culturing aliquots from the BICCP on fresh agar-minimal medium and let grow. After 5 days, the grown culture colonies were photographed and counted. Data were calculated and compared relative to the control cultures, supplemented with no calcium and performed no precipitations.

### Conversion efficiency percentage (CE%)

CE% deals with the percentage of calcium carbonate formed relative to the calcium salt given to the bacterial cells in a culture. It was calculated as percentage of Kg CaCO_3_ produced/ Kg calcium salt given per 10^6^ cells as follows:

1 $${\text{CE}}\% \, = {\text{ Weight of CaA }}*{1}00/{\text{Weight of CaCO}}_{{3}} = {\text{ X}}\% /{1}0^{{6}} {\text{cells}}$$

#### Carbon capture and sequestration calculation

This item deals with carbon dioxide removal from the atmosphere during bacterially induced CaCO_3_ precipitation. CO_2_ is incorporation and sequestration in BICCP throughout the life span of the bacterial cells takes place according to the following equation:

2 $${\text{Ca}}^{{{\text{2}} + }} {\text{A }} + {\text{ CO}}_{{\text{2}}} + {\text{ H}}_{{\text{2}}} {\text{O}} \to {\text{CaCO}}_{3}^{ - } \downarrow + {\text{ H}}_{{\text{2}}} {\text{A}}$$

‘’A’’ is the anion of calcium salts given whether citrate “Cit”, acetate “Ac”, chloride “Cl” or nitrate “Cl”. The above equation shows that conversion of the different calcium salts to calcium carbonate fixes CO_2_ in equal molar ratios (mole CO_2_/mole CaCO_3_). This molar relationship in Eq. (2) is utilized to calculate carbon capture via the process of BICCP by *B. subtilis,* as:

$${\text{Carbon captured and sequestered }} = {\text{ mole CO}}_{{2}} {\text{fixed }} = {\text{ mole CaCO}}_{{3}} {\text{formed}}$$

The weight of CaCO_3_ is calculated as moles, which equals the moles of CO_2_ removed from the atmosphere.

### Economic feasibility

The environmental impact of bacterially-induced calcium carbonate (BICCP) in CDR (CO_2_ removal) from the atmosphere has been correlated to economic feasibility and costs. The economic feasibility of utilizing MICP in CDR has been calculated in different terms.

1. Economic feasibility 1 (EF1) of BICCP in CDR as moles CO_2_ per Kg calcium salt given.
2. Economic feasibility 2 (EF2) of BICCP in CDR as moles CO_2_ fixed/ US$.
3. Economic feasibility 3 (EF3) of BICCP in CDR as US$c/m^3^ atmosphere, considering the atmospheric CO_2_ concentration is 0.04%.

### Estimation of field capacity and characterization of *Vicia faba*

A definite weight of sand soil was placed in glass tubes with two open ends, sealed at one of the ends by gauze, filled with water, let for 24 h, reweighed thereafter. Field capacity of the sand soil sample was calculated according to the following equation according to Schulte and Hopkins^24^.

$${\text{Field capacity }}\% \, = \, \left( {{\text{Wet soil weight }}{-}{\text{ Dry soil weight}}} \right)*{ 1}00/{\text{dry soil weight}}$$

The above procedure was repeated, replacing water with the bacterium, nutritive media, and the optimum calcium salt concentration (10 g/l CaCl_2_), to induce calcium carbonate formation. The last procedure aims to identify the changes that might take place in the field capacity as a result of calcium carbonate formation.

Calcium carbonate content, synthesized by bacterial isolate, at the top and bottom of the same column of sand soil was estimated by the method of Tiessen et al.^25^. A soil sample (1 g) was treated with HCl (0.5 N) and the residual was titrated against NaOH (0.5 N) and carbonate content was calculated by the following equation:

$${\text{Carbonate }}\left( \% \right) \, = \, ({25 }\left( {\text{mL HCl}} \right) \, - {\text{ V }}\left( {\text{ml NaOH}} \right) \, * \, 0.0{15}*{ 1}00/{\text{1g }}\left( {\text{weight of soil}} \right) \, = {\text{ X}}\%$$

This soil was then used for germinating *V. faba* seeds and growth of their seedlings for 7 days. Seeds of *V. faba* were germinated at the modulated field capacity by the CaCO_3_ formed by *B. subtilis*.

### Adsorption of heavy metals

Adsorption of heavy metals from sewage effluent before and after 3 days at BICCP (bacteria, urea, calcium salts were included) as follows:

3 $${\text{SHM }} = \, \left( {\left[ {{\text{hm}}} \right]_{{{\text{before}}}} - \, \left[ {{\text{hm}}} \right]_{{{\text{after}}}} } \right)*{1}00/\left[ {{\text{hm}}} \right]_{{{\text{before}}}}$$

SHM is adsorbed heavy metals.

### Healing concrete cracks

Artificial cracks were made in petri dishes. Some sand was thoroughly mixed with some cement and then watered in petri dishes. Petri dishes were placed in oven at a temperature of 120 °C for 30 min; then left open to cool down at laboratory conditions, plates got cracks. The healing cocktail, at its proper concentrations (minimal medium, bacteria (561 * 10^8^ cell/mL), optimum salt and concentration of 15 CaCl_2_ g/L and 20 g/L urea) was poured every other day for 2 weeks on the developed cracks.

### Statistical analysis

Each experiment was performed in triplicate, and the results are presented as mean values ± standard error (SE). Statistical analysis was conducted using one-way ANOVA (Analysis of Variance) in SPSS version 21, and Duncan’s multiple range test was used to determine significant differences at the 0.05 significance level.

Two-way ANOVA was carried out to achieve the effect of time and different concentrations of calcium salts and their interaction on different parameters estimated in *B. subtilis* and eta square ‘‘η^2^’’ was calculated as: ‘‘η^2^’’ = SSEffect/ SSTotal to achieve the size effects of each factor or the interaction between factors. In addition, Pearson correlation analysis was performed to obtain the relation between some parameters.

## Results

**Fig. 1:**
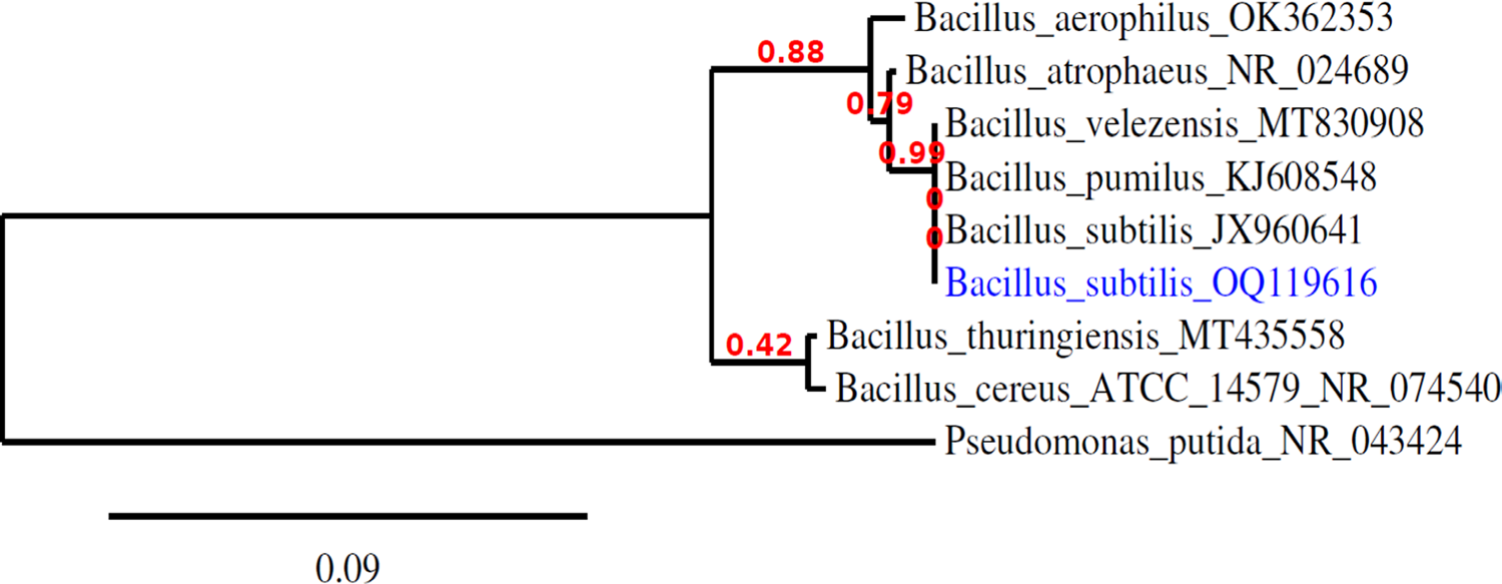
Phylogenetic tree on the basis of patterns and genetic relationship of the case-studied *Bacillus subtilis* OQ119616. (For interpretation of the references to color in this figure legend, the reader is referred to the Web version of this article.

**Fig. 2:**
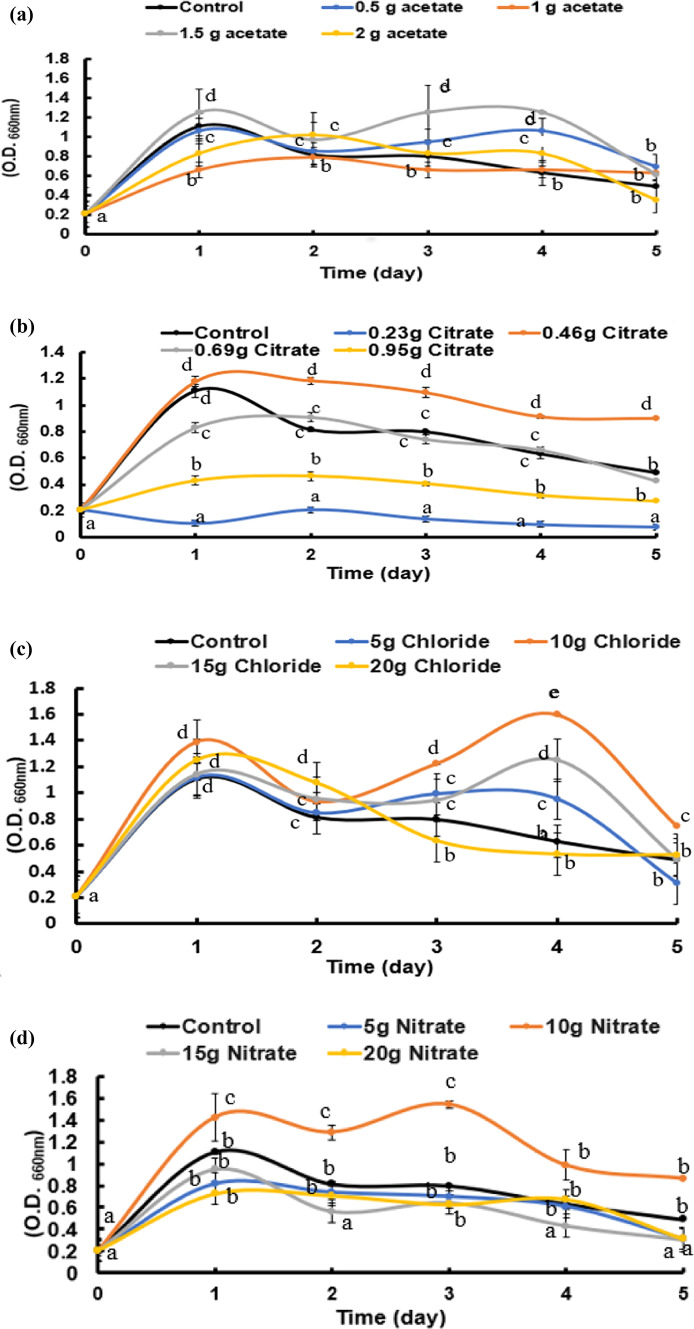
Growth of *Bacillus subtilis* as optical density (O.D._660 nm_) was followed over a period of 5 days at minimal medium supplemented with four levels of four calcium salts (**a**: acetate, **b**: citrate, **c**: chloride, and **d**: nitrate). Cultures started at 0-time with O.D._660 nm_ of 0.2A accounting to 21 × 10^7^ cells/mL. Data are means ± SE, n = 3. At each day, means with different letters are significantly differ at *P* ≤ 0.05 according to Duncan’s test.

**Fig. 3:**
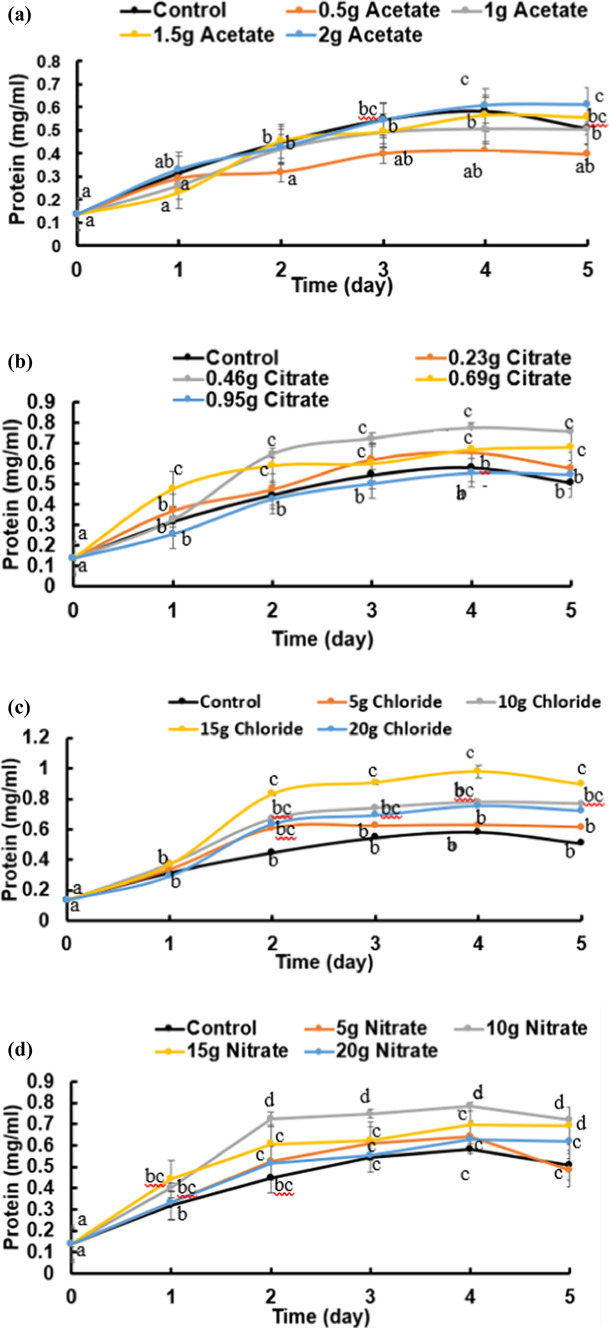
Protein content of *Bacillus subtilis* (mg.ml^-1^) was followed over a period of 5 days at minimal medium supplemented with four levels of four calcium salts (**a**: acetate, **b**: citrate, **c**: chloride and **d**: nitrate). Cultures started at 0-time with O.D._660 nm_ of 0.2A accounting to 21 × 10^7^ cells/mL. Statistical analysis as shown in Fig.  2.

Figure 1 shows the phylogenic tree of the bacterial strain, which has been locally isolated from Sahel Seleem city, Assiut Governate, Egypt. It has been purified, characterized phenotypically and genetically identified by molecular approaches using 16S technology as *B. subtilis* OQ119616; it is used in this study to precipitate CaCO_3_. Figures 2 and 3 shows the growth curves (OD_660nm_ and protein contents) of *B. subtilis* over a period of 5 days in minimal medium containing only peptone and beef extract but supplemented with successively increasing concentrations of calcium chloride “Cl”, calcium citrate “Ci”, calcium acetate “ac” or calcium nitrate “N”, in addition to urea in concentrations stated in “Materials and Methods”. Culture started at 0-time with O.D._660 nm_ of 0.2A accounting to 21 × 10^7^ cells/mL. The growth magnitude and rate were significantly increased at 10 g/L CaCl_2_ while the lowest growth was at 0.2 g/L calcium citrate. The maximum growth was on day 4.

Calcium citrate and Calcium chloride showed a highly significant effect (*p* < 0.001), with an F-value of 165.464 and 1679.909; respectively indicating a strong influence of calcium citrate and CaCl_2_ concentration on the growth parameter (OD). Also, calcium nitrate showed a statistically significant effect (*p* < 0.001), with an F-value of 22.712, indicating notable but less extreme variation compared to CaCl_2_ and Ca-citrate (Table S1).

The ANOVA analysis revealed statistically significant differences, all calcium salt treatments resulted in statistically significant changes (*p* < 0.05), with CaCl_2_ showing the strongest influence, followed by Ca-citrate, Ca-acetate, and Ca-nitrate on protein content of *B. subtilis* (Table S2).

**Fig. 4:**
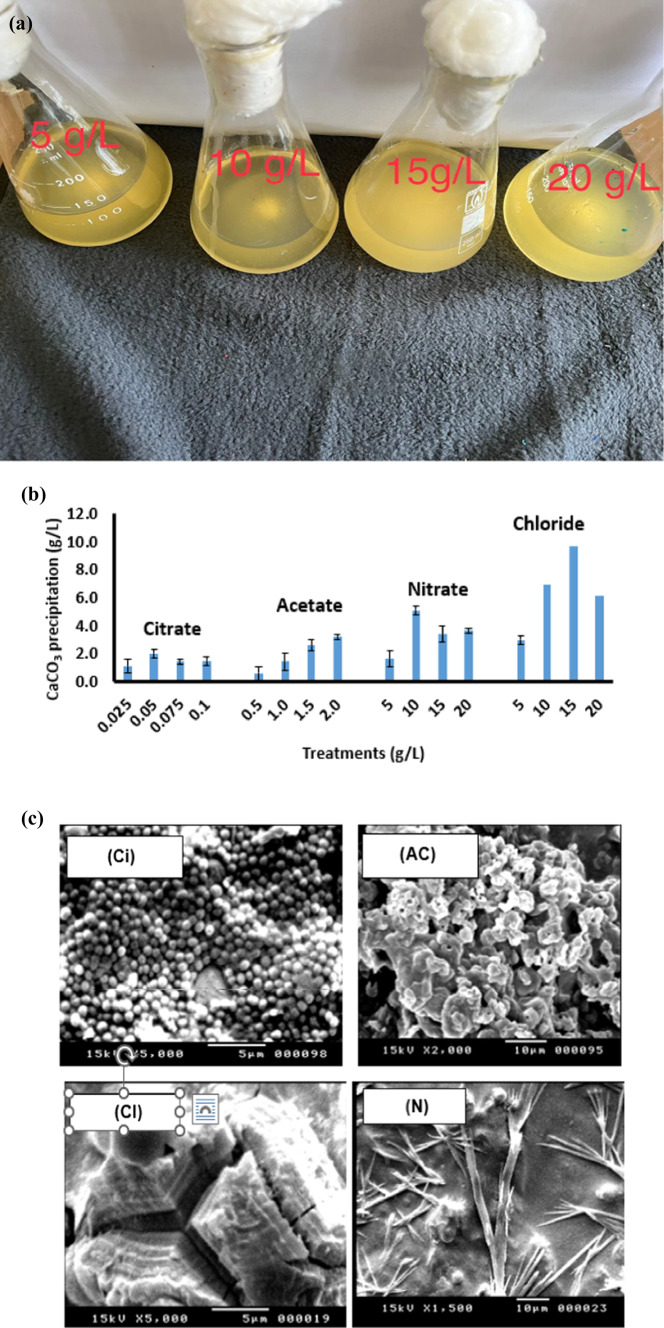

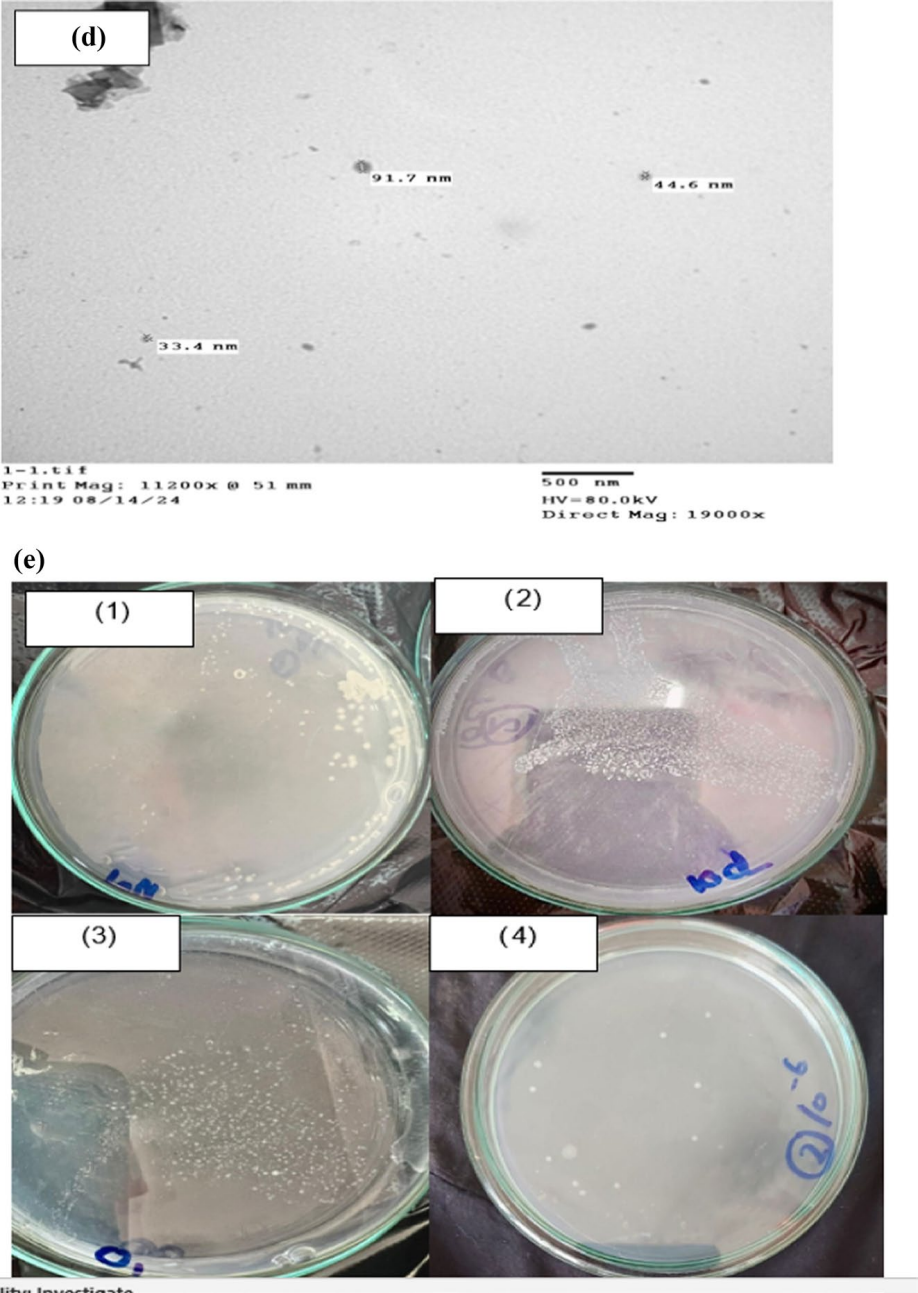
Calcium carbonate precipitation by *Bacillus subtilis*(g/L) after growth for 30 days in minimal medium supplemented with four levels of four calcium salts (citrate “Ci” acetate “Ac”, nitrate “N” and chloride “Cl”) (**a**); development of calcium carbonate precipitation over time of 30 days at successively increasing concentrations of CaCl_2_ (**b**); Scanning electron micrographs (SEM) showing shapes and morphogenesis of calcium carbonate precipitations produced by *Bacillus subtilis* at the provided calcium salts (**c**) [4cCi, 4cAc, 4cN and 4cCl for citrate, acetate, nitrate, and chloride, respectively]; (**d**) transmission electron micrographs (TEM) showing nanoparticles of calcium carbonate precipitations produced by *Bacillus subtilis* at the provided calcium chloride and viability of bacterial cells in calcium carbonate precipitates (**e**1-4) and.

Throughout its growth period in minimal medium supplemented with any of the four levels of the four calcium salts, *B. subtilis* exhibited its ability to precipitate CaCO_3_; calcium carbonate precipitates were developing gradually over time up to their maximum on day 30th. Figure 4a shows the maximum developed calcium carbonate precipitation; turbidity can be noticed visually as indicative for concentrations**.** The amounts of CaCO_3_ precipitation are presented as g/L in Fig. 4b; the different calcium salts induced calcium carbonate formation at variable quantities depending on the type and concentration of the calcium salt given to *B. subtilis* in the culture medium in the following order: chloride > nitrate > acetate > citrate. The concentration of 15 g/L calcium chloride resulted in a significant increase in production/precipitation of CaCO_3_ (9.7 g/L) while the lowest amount was obtained at 0.5 g calcium acetate (0.6 g/L).

The calcium carbonate produced by *B. subtilis* showed different morphologies, at the different calcium salts provided to the bacterium (Fig. 4c). Scanning electron micrographs (SEM images) demonstrate the different morphs shapes of BICCP as spherical in calcium citrate (Fig. 4c Ci), irregular in calcium acetate (Fig. 4c Ac), needle in calcium nitrate (Fig. 4c Ac) and rhombohedral for chloride (Fig. 4c N). Figure 4d showing nanoparticles of calcium carbonate precipitations produced by *B. subtilis* at the provided calcium chloride the bacterial cells remained living using transmission electron micrographs (TEM).

After the 30 days growth in such CaCO_3_-rich medium (Fig. 4e), the viability of cells is manifested by their recommencement of growth after transfer in a new fresh medium. Sustained viability is essential for enduring BICCP process and, meanwhile indicates their tolerance to harsh conditions of CaCO_3_ for a long period of time.

**Fig. 5:**
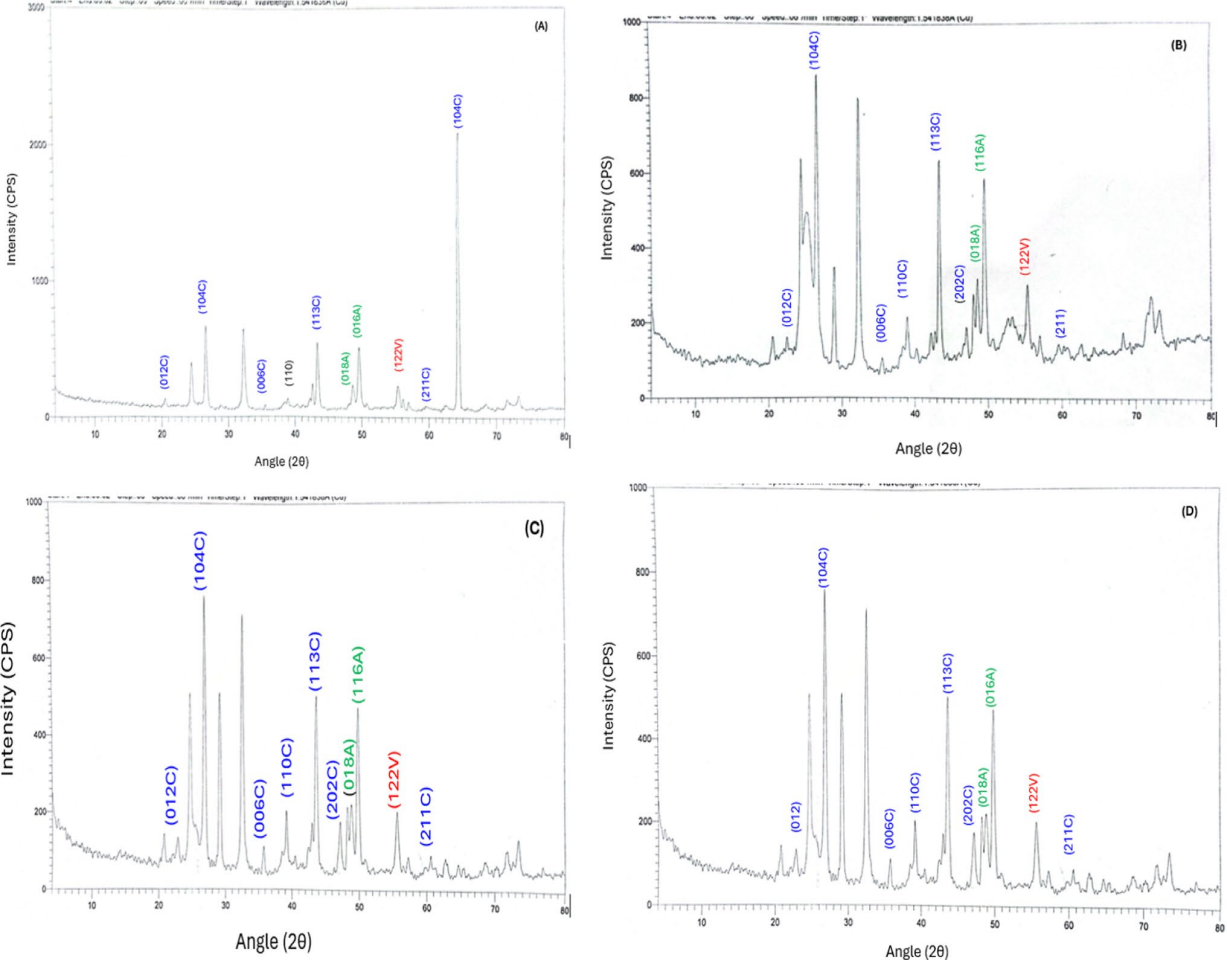
XRD pattern of Calcium Carbonate (**A**): acetate, (**B**): citrate, (**C**): chloride, (**D**): nitrate. X-ray diffraction patterns of calcite CaCO_3_, vaterite CaCO_3_ and aragonite CaCO_3_ polymorphs obtained by biomineralization of *Bacillus subtilis* culture after 5 days of exposure.

Figure 5 shows the XRD patterns of the four polymorphs of calcium carbonate crystals. The patterns match those of X-ray diffraction standards. The XRD spectrum was compared with that of calcite, vaterite, and aragonite standards (JCPDS card number 47-1743). It is evident that all four calcium carbonate polymorphs were synthesized as expected with some impurities being observed.

**Fig. 6:**
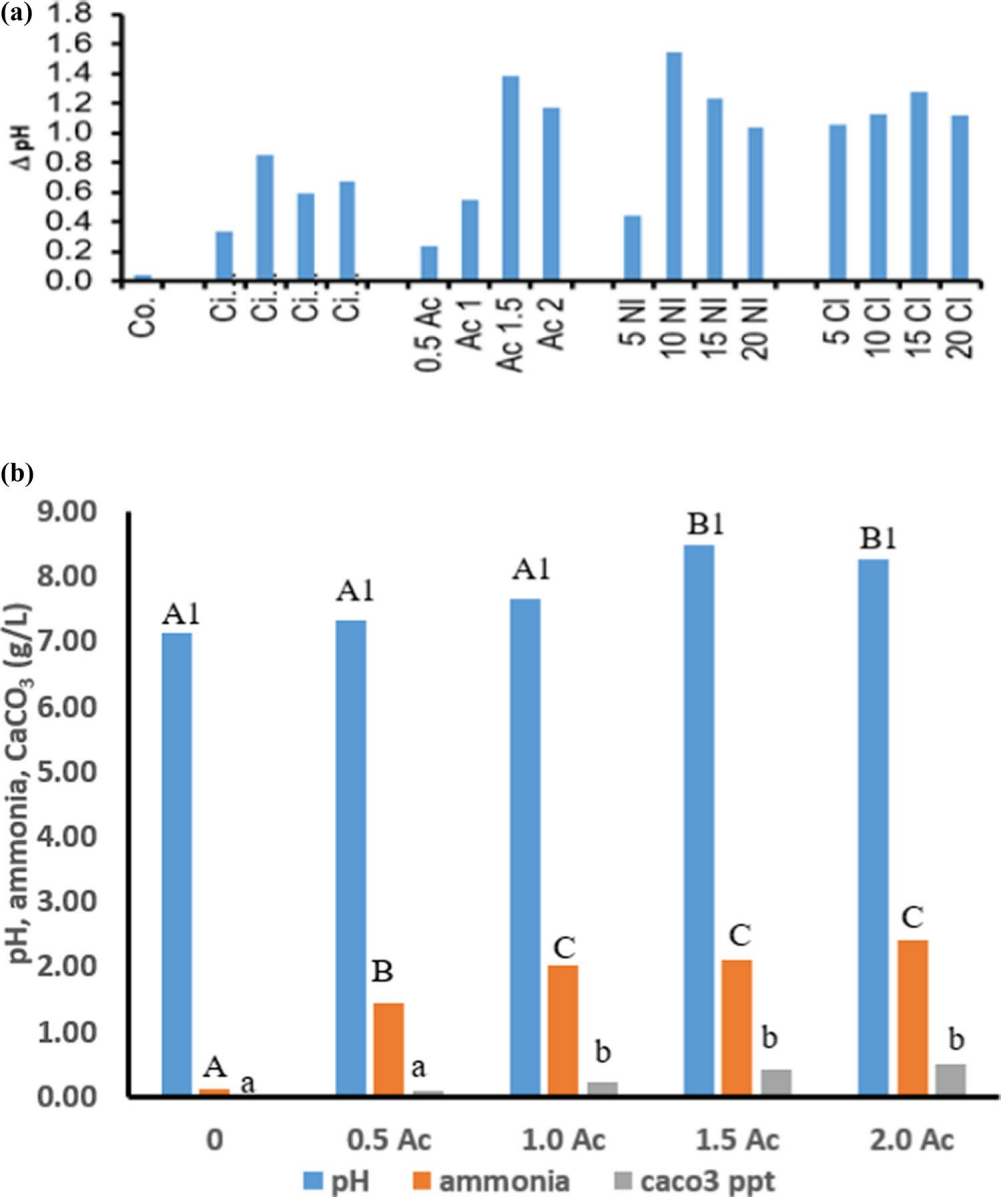
ΔpH changes relative to pH 7.1 (control) (**a**), relationship between acetate concentrations and pH, ammonia and CaCO_3_ produced (**b**),

As MICP is strongly dependent on the pH of the medium, the final pH values at the end of the growth period (30 days) were recorded in the variously treated cultures of *B. subtilis*. Figure 6a presents the changes (Δ pH) in the pH values relative to 7.1; the pH of control cultures that received none of the treatments (neither calcium nor urea). The Δ pH of the culture media was modulated to the alkaline side and exhibited dependence on the type of calcium salt and concentration. Citrate induced the least elevation in Δ pH (0.3–0.8), acetate (0.2–1.4), nitrate (0.4–1.5) and chloride (1.1–1.3), i.e. in the following order: acetate > nitrate > chloride > citrate i. e. acetate induced the highest Δ pH shifts relative to the control. pH increased significantly with increasing acetate concentration up to 1.5 g/L. Similarly, ammonia content and CaCO₃ precipitation followed the same trend as pH (Fig. 6b).

**Fig. 7:**
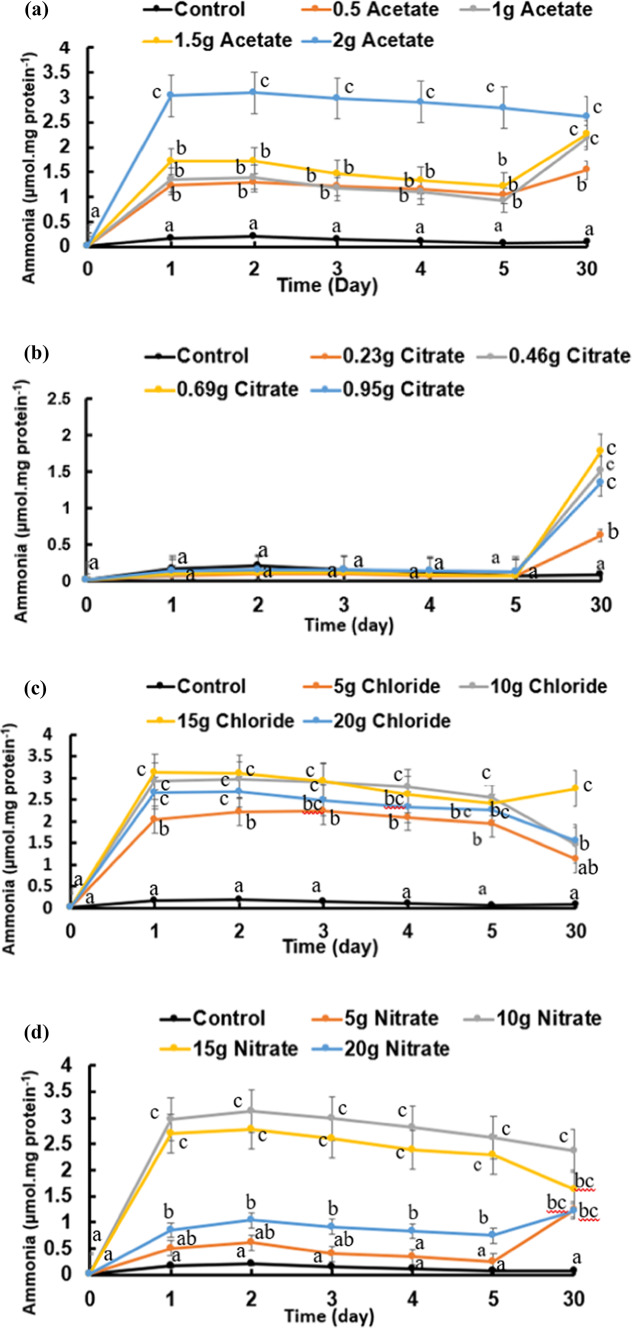
Ammonia content of *Bacillus subtilis* cultures assessed over a period of 5 days at minimal medium supplemented with four levels of four salts of calcium (**a**: acetate, **b**: citrate, **c**: chloride and **d**: nitrate). Cultures started at 0-time with O.D._660 nm_ of 0.2A accounting to 21 × 10^7^ cells/mL. Statistical analysis as shown in Fig. 2.

**Fig. 8:**
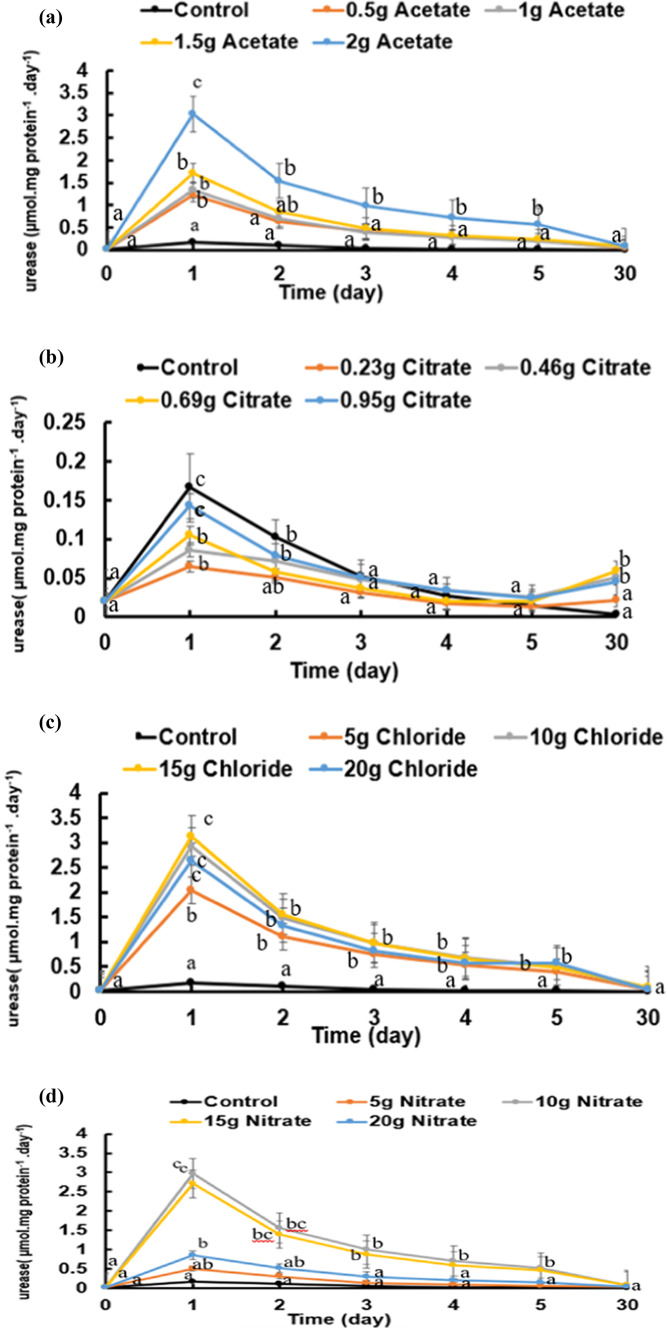
Urease activity of *Bacillus subtilis* was assessed as ammonia over a period of 5 days at minimal medium supplemented with four levels of four salts of calcium (**a**: acetate, **b**: citrate. **c**: chloride and **d**: nitrate). Cultures started at 0-time with O.D._660 nm_ of 0.2A accounting to 21 × 10^7^ cells/mL. Statistical analysis as shown in Fig. 2.

**Fig. 9:**
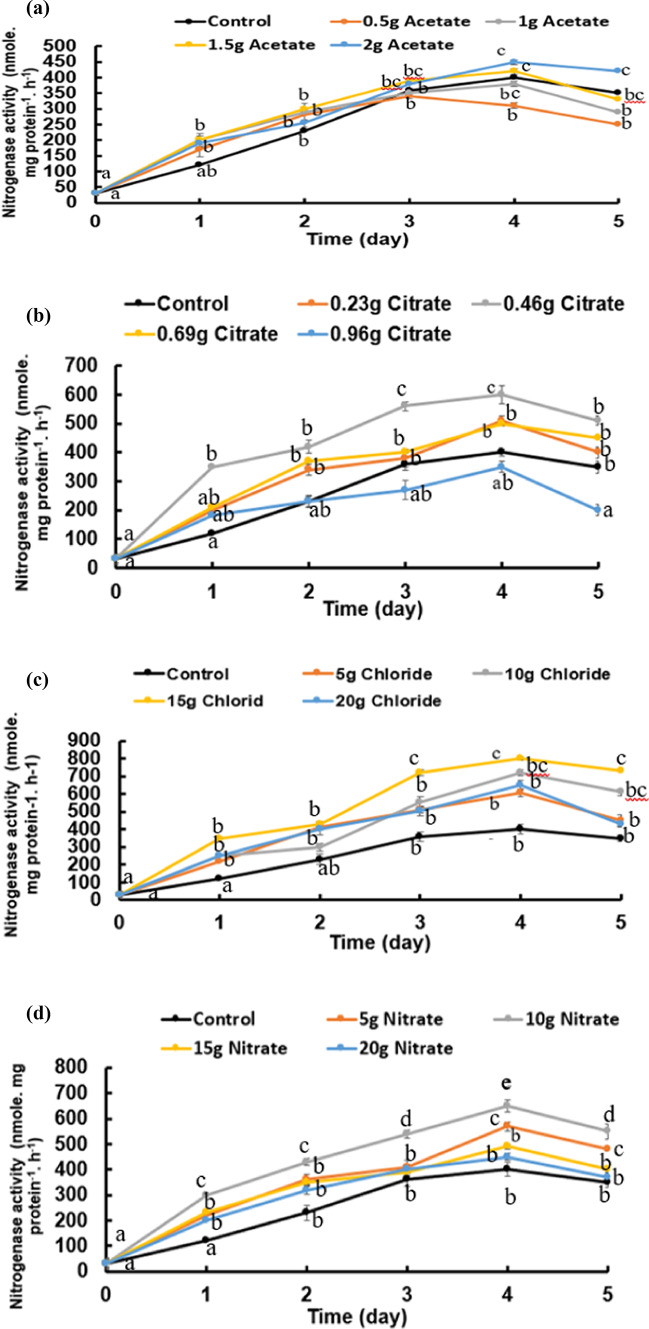
Nitrogenase activity of *Bacillus subtilis* was assessed as ammonia over a period of 5 days at minimal medium supplemented with four levels of four salts of calcium (**a**: acetate, **b**: citrate, **c**: chloride and **d**: nitrate). Cultures started at 0-time with O.D._660 nm_ of 0.2A accounting to 21 × 10^7^ cells/mL. Statistical analysis as shown in Fig. 2.

As the above-described pH shifts to the alkaline side are strongly dependent on ammonia, its contents were followed in the variously treated cultures of *B. subtilis* and presented in Fig. 7a–d. As a general notice, ammonia contents of most cultures attained their significantly highest pH values on day 1 and sustained thereafter; it was mostly unchanged. However, only citrate concentrations sustained low pH values during the period of active growth (the first 5 days) but attained higher pHs by the end of the experiment (day 30). Although the added calcium salts elevated the pH of the cultures relative to the control cultures, the elevation of the pH was not proportional with the calcium salt concentration given. As ammonia results mainly from the activity of the urease enzyme, (Fig. 8a–d) shows the urease activity of *B. subtilis*, assessed daily by as µmole ammonia.mg DW t^−1^_._ d^−1^., over a period of 5 days growth at minimal medium supplemented with four levels of four salts of calcium (chloride, citrate, acetate, and nitrate). Final ammonia contents of the cultures were assessed at the end of carbonate synthesis i.e. day 30. The activity was 0.16 µmole ammonia.mg DW t^−1^ d^−1^ at control cultures supplemented with neither calcium nor urea (Fig. 8). Citrate, however, induced the least values of urease compartive to the other salts and less than the control (Fig. 8b). Calcium nitrate (Fig. 8d) at a concentration of 10 g/L induced significantly the highest enzyme activity (2.97 µmol mg protein^−1^ day^−1^) while calcium chloride (Fig. 8c) at 15 g/L exhibited the highest rate (about 3.1 µmol mg protein^−1^ day^−1^). Nitrogenase activity catalyzes biological nitrogen fixation (BNF) as atmospheric nitrogen is converted to ammonia. Nitrogenase activity is usually assessed by the acetylene reduction technique, which is equivalent to ammonia in nature. The highest significant rate (800 nmole. mg protein^−1^ h^−1^) at a concentration of 15 g/L calcium chloride lowest activity (350 nmole. mg protein^−1^ h^−1^) at a concentration of 0.96 calcium citrate (Fig. 9a–d). The enzyme activity increased significantly at day 4 for all salts.

In summary, the ANOVA analysis showed that all calcium salt treatments significantly influenced the measured outcome, with Ca-nitrate and Ca-acetate showing the most pronounced effects. These results highlight that the type and concentration of calcium salts distinctly affect the biological or chemical response being studied (e.g., ammonia production, urease activity, CaCO₃ precipitation and nitrogenase activity) (Tables S3, S4, S5 and S6).

**Fig. 10:**
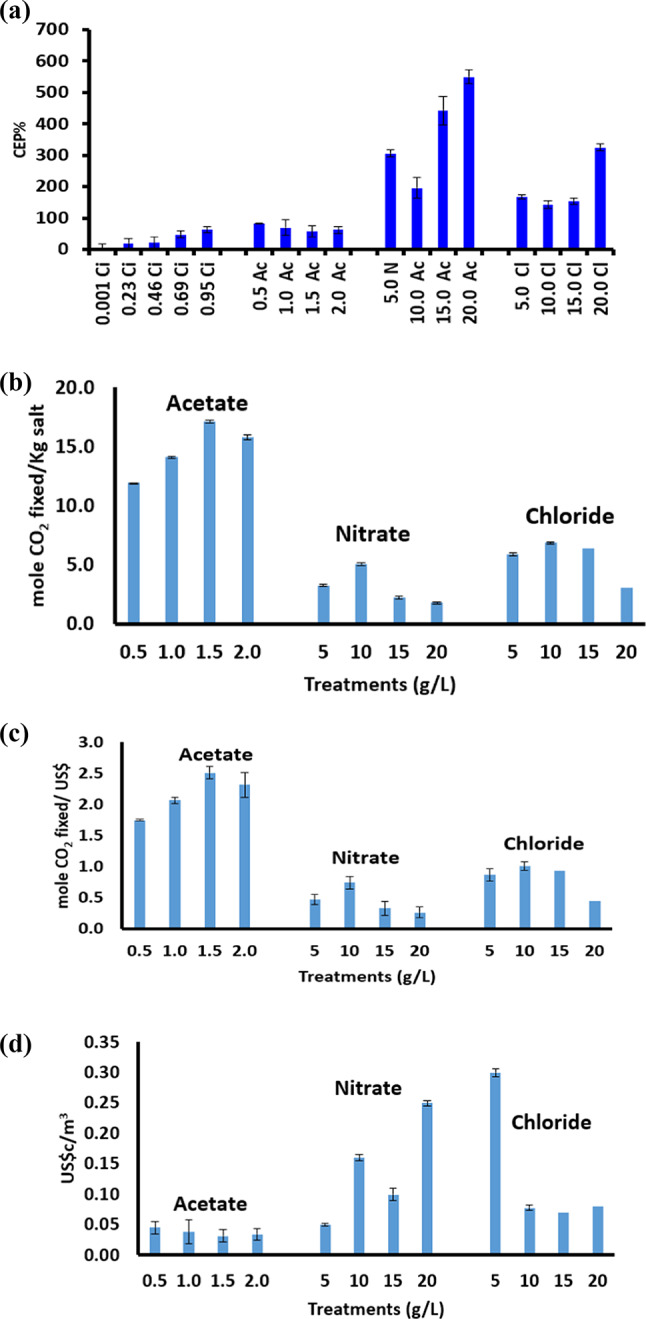
Conversion efficiency percentage (CE%) of the given calcium salts to calcium carbonate formed (**a**); Economic feasibility 1 (EF1) of BICCP in CDR as (moles CO_2_ fixed/Kg salt given (**b**); Economic feasibility 2 (EF2) of BICCP in CDR as moles CO_2_ fixed/ US$ (**c**1); Economic feasibility 3 (EF3) of BICCP in CDR as US$c/m^3^ atmosphere (**c**2).

The conversion efficiency percentage (CE%) of the various calcium salts given to *B. subtilis* relative to the CaCO_3_ produced (CE% = weight of calcium salt given*100/weight of CaCO_3_ precipitated = X%/Y10^6^ cell) exhibited an attitude different from absolute quantities (Fig. 9a). The highest conversion efficiency percentage (CE%) of 550% was induced at nitrate while the lowest CE% of 21% was found at citrate. Citrate was accompanied by the lowest amounts of carbonate precipitation among the other salts; particularly 0.6 g/L was obtained at 0.5 g calcium citrate (Fig. 10a). The biggest amount of 9.6 g/L CaCO_3_ precipitation was obtained at 15 g/L calcium chloride; increasing concentration of CaCl_2_ up to 20 g/L did not induce further enhancement of CaCO_3_. As CO_2_ is fixed into CaCO_3_ in mole/mole ratios during BICCP according to the following formula: Ca^2+^  + CO_2_ + H_2_O → CaCO_3_; this trait can be utilized purposefully in carbon dioxide removal (CDR) from the atmosphere. The economic feasibility of utilizing MICP in CDR has been calculated in three terms. First, is Economic feasibility 1 (EF1) of BICCP in CDR as moles CO_2_ per Kg calcium salt given moles CO_2_ fixed/Kg salt (Fig. 10b), second is Economic feasibility 2 (EF2) of MICP in CDR as moles CaCO_3_ per Kg calcium salt given moles CO_2_ fixed/ US$ (Fig. 10c1) and third is Economic feasibility 3 (EF3) of BICCP in CDR as US$c/m^3^ atmosphere, taking into account atmospheric CO_2_ concentration as 0.04% (Fig. 10c2). In this regard, acetate can be considered the most efficient salt in CO_2_ capture and sequestration as it exhibited the least expensive salt in relation to the amount of CO_2_ fixed (precipitated). Once again, calcium citrate, although exhibited the highest conversion efficiency, would be excluded from consideration in CO_2_ sequestration and removal as its solubility is scarce, which results conclusively in trivial quantities.

In addition to carbon capture and sequestration, some other applications were undertaken. Field capacity (the maximum water holding capacity) is used to assess alterations in soil characteristics concerning water economics. In this work, a sand soil column of 25 cm height. Supplemented with *B. subtilis*, CaCl_2_ and urea, this cocktail was used for evaluating the impact of the formed BICCP on the field capacity of a sand sample. The data of field capacity alterations are presented in Table 1; the results show that the field capacity of a sand soil column was elevated, indicating that the formation of CaCO_3_ was accompanied with improvement in soil characteristics to hold more water. The sand column was divided into top and bottom sand samples (12.5 cm and 12.5 cm, respectively). The bacterially-induced calcium carbonate by *B. subtilis* (BICCP) has improved the field capacity of sand soil, higher 150% in top soil and 30% in bottom soil than the sand control (without BICCP) at a sand column of 25 cm high. The lower part of the sand column exhibited less field capacity than the upper part, due to gravitational effect. The elevation of the filed capacity stimulated the germination of *V. faba* seeds and the length of their plumules (Table 1).

**Table 1:** Field capacity of a sand soil sample as influenced by BICCP (*Bacillus*-induced calcium carbonate precipitates) and its consequences on *Vicia faba* seeds germination and plumule length in 7 days.

Soil treatments	C1		C2		C3		C4	
	Top	Bottom	Top	Bottom	Top	Bottom	Top	Bottom
CaCO_3_ precipitated (%)	2.25	1.75	3.75	3.75	2.75	1.75	4.9	6.25
Field capacity (%)	4.1	14.66	6.6	17.5	5.4	16.7	10.5	20.26
Number of germinated seeds	1.0	0.0	1.0	1.0	2.0	0.0	2.0	4.0
Plumule length (cm)	0.4	0.0 ± 0	1.2 ±	1.5 ±	1.1 ±	0.0 ±	1.0 ±	1.85 ±

*C is the controlling treatments, C1 is distilled water, C2 is minimal medium + urea + CaCl_2_ and C3 is minimal medium and bacteria, C4 is minimal medium + bacteria (*B. subtilis*) + urea + CaCl_2_, Top and Bottom are the upper and lower parts of the sand column, respectively.

Second, BICCP was also used to adsorb heavy metals from sewage effluents for partial cleansing of waste/sewage effluent from contaminants for environment, hygiene and reuse. BICCP induced adsorption of some mineral elements (heavy metals); namely, Pd, Zn, Cd and Cu. Heavy metals concentrations (Pd, Zn, Cd and Cu) were assessed before and after treatment with *B. subtilis* in the presence of BICCP requirements (CaCl_2_ and urea at their optimum concentrations). Mineral adsorption has been proven to occur as MICP is formed. The results revealed a sharp decrease in soluble heavy metal levels in the effluent (Table 2).

**Table 2:** Adsorption of some heavy metals (SHM) by BICCP (*Bacillus*-induced CaCO_3_).

Heavy metals	Amounts removed (mg/ml)	Removal percentage
Cd	35	40
Pb	32	70
Cu	40	55
Zn	21	25

Contents of heavy metals before (original at 0-time) and after (residual after 7 days) adsorption from sewage effluent were subtracted to give amounts removed; removal percentages were calculated as the percentage of residual amounts relative to the originals (number of samples n = 3).

**Fig. 11:**
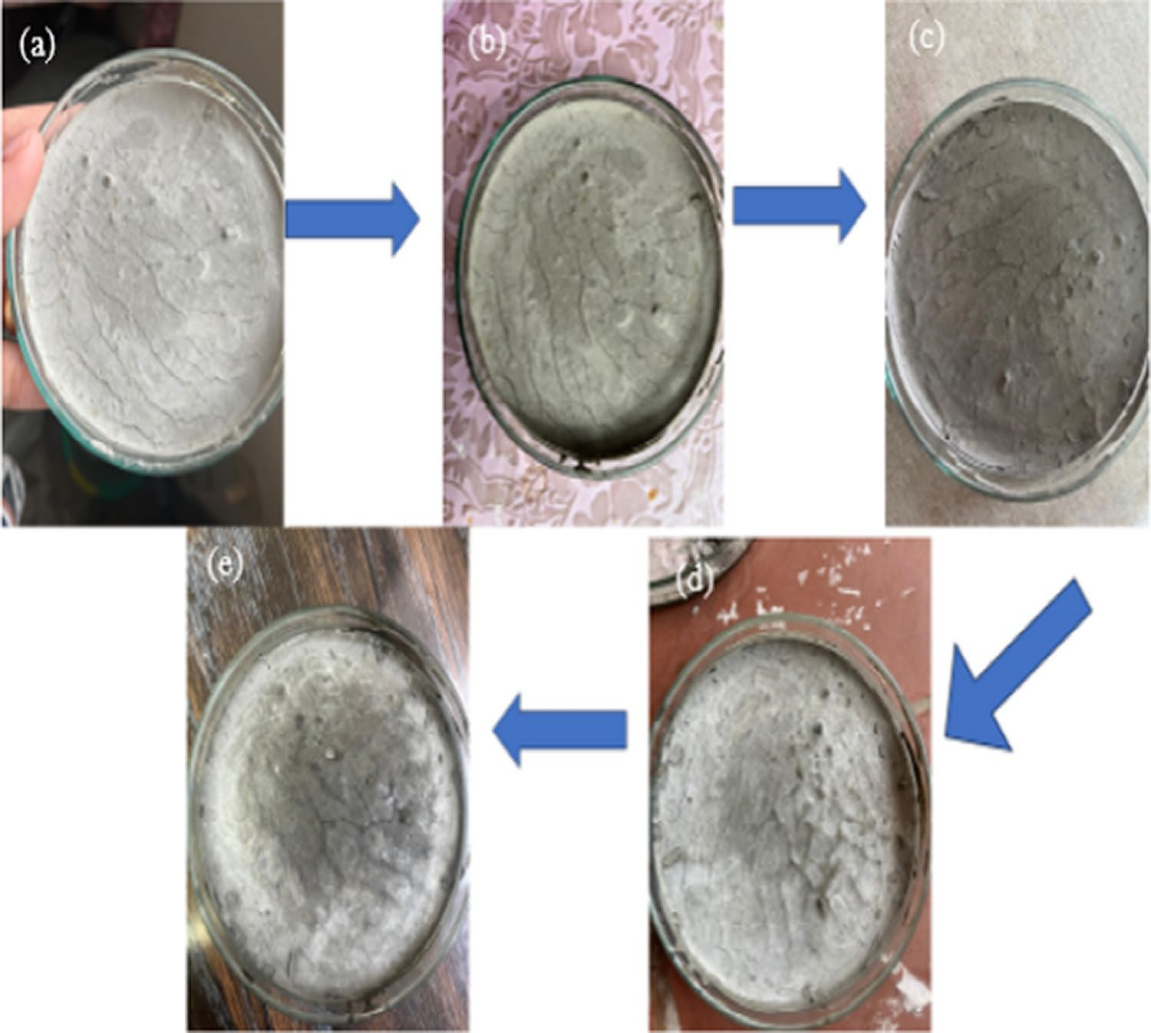
Concrete cracks healing by BICCP; progression of cracks healing is demonstrated as paces in figure (**a**–**e**); developed cracks (**a**), added healing cocktail (**b**), healing progress (**c** & **d**), healed cracks.

Third, BICCP was tested to see if it could repair artificially induced concrete cracks in petri dishes of 1-2 mm wide and 10 mm long (Fig. 11a). Cracks were treated every other day with the healing cocktail (CaCl_2_ and urea at their optimum concentrations) were completely healed by the precipitated CaCO_3_ with 561 * 10^8^cells/mL in 14 days. Figure 11b-d shows progression of healing while Fig. 11e the completely healed cracks.

Correlation between various growth criteria and enzymes activity with different concentrations of calcium salts of *B. subtilis* was estimated. Table 3 showed that there is a weak positive correlation between growth parameters (OD and protein) and calcium acetate concentrations and a negative weak correlation for the other salts with OD. There is a strong positive correlation between CaCO_3_ ppt and all salts concentration except calcium citrate which gave a weak negative correlation. Also, there is a strong positive correlation between ammonia content and urease activity with acetate and chloride salts. Nitrogenase activity gave a weak positive correlation with most salts.

**Table 3:** Correlation between various growth criteria and enzymes activity with different concentration of calcium salts of *Bacillus subtilis* grown for 5 days at successively increasing levels of calcium salts.

Calcium salts	O.D	Protein	CaCO_3_ ppt	Ammonia content	Urease activity	Nitrogenase activity
Acetate	0.153	0.418	0.772	0.927*	0.938*	0.161
Citrate	− 0.085	0.037	− 0.052	0.101	− 0.004	0.037
Chloride	0.178	0.685	0.772	0.761	0.788	− 0.261
Nitrate	− 0.224	0.381	0.610	0.451	0.446	0.569

The positive sign (+) indicates positive correlations and the negative sign (−) indicates negative correlations while one star (*) indicates weak correlation value (< 0.7) and two stars (**) indicate strong correlation (≥ 0.7); no star indicates no correlation.

Protein gave a significant strong positive correlation with time for all salts. Ammonia content gave a positive range from weak to strong correlation with time. Urease activity gave a weak negative correlation with time to all salts. Nitrogenase activity gave a significant strong positive correlation with time to all salts (Table 4).

**Table 4:** Correlation between various growth criteria and enzymes activity with different calcium salts of *Bacillus subtilis* grown for 5 days at successively increasing levels of Time.

	Calcium salts		O.D	Protein	Ammonia content	Urease activity	Nitrogenase activity
Time	Ac	0.50	0.41667	0.886885*	0.532895	− 0.264633	− 0.266502
		1.00	0.52536	0.919315**	0.379146	− 0.300228	0.740715
		1.50	0.28686	0.935375**	0.390579	− 0.297238	0.799242
		2.00	0.08724	0.955662**	0.592404	− 0.243035	0.835985*
	Cit	0.23	− 0.70081	0.880513*	0.429939	− 0.505793	0.948661**
		0.46	0.37485	0.909954*	0.766855	− 0.306067	0.886985*
		0.69	0.08480	0.866760*	0.253276	− 0.431807	0.844762*
		0.95	− 0.03144	0.943370**	0.508702	− 0.381078	0.913322*
	Cl	5.00	0.02353	0.846088*	0.610423	− 0.231668	0.700877
		10.0	0.38292	0.899979*	0.560328	− 0.250310	0.927218*
		15.0	0.23224	0.877540*	0.463238	− 0.275708	0.822274**
		20.0	− 0.13667	0.902228*	0.526992	− 0.228879	0.923089**
	Nit	5.00	− 0.02585	0.772414	0.118963	− 0.365693	0.897497*
		10.0	0.24491	0.838640*	0.558867	− 0.249678	0.874564**
		15.0	− 0.19642	0.883894**	0.522130	− 0.261994	0.831261*
		20.0	0.06764	0.924655**	0.509695	− 0.250989	0.826933*

The positive sign (+) indicates positive correlations and the negative sign (−) indicates negative correlations while one star (*) indicates weak correlation value (< 0.7) and two stars (**) indicate strong correlation (≥ 0.7); no star indicates no correlation.

Data of two-way ANOVA revealed a significant increase in all parameters (OD, protein, ammonia content, urease and nitrogenase activity) with time, calcium salts and the interaction between time and calcium salts.

To measure the size effect of each factor, η^2^ was calculated by dividing the sums of squares for the effecting factor (SSeffect) on the total sums of squares for all effects including errors and interactions (SStotal) in the ANOVA. Values of η^2^ in Table 5 showed that using calcium acetate, calcium citrate and calcium nitrate exerted the large magnitude (strong) of effect on the changes of growth parameter (OD) in *B. subtilis*, with increasing salt concentrations except in the case of calcium chloride give a small effect with concentration of salt and gave a large magnitude with time and interaction of time and salt concentrations. Protein values revealed that the effect of time has induced a large magnitude for all calcium salts. In contrast, the salt concentrations gave a moderate to large magnitude with ammonia content for all salts. The interaction between time and salt concentrations gives a high magnitude of effect on the changes of nitrogenase activity with all salts. The size effect is based on commonly accepted benchmarks (small ≥ 0.01, moderate  ≥ 0.06, large ≥ 0.14).

**Table 5:** The eta squared (η^2^) for the magnitude of effect of Calcium salt concentrations or the time and the interaction between them on changes of each studied parameter in *Bacillus subtilis.*

Salts	Source	OD		Protein		Ammonia content		Nitrgenase activity	
		η^2^	F-value	η^2^	F-value	η^2^	F-value	η^2^	F-value
Acetate	Time	0.15 L	3.62*	0.637L	76.87*	0.016S	51.8*	0.015S	98.62*
	Ac	0.21 L	5.1**	0.183 L	22.06*	0.974 L	2995.6*	0.044S	280.11*
	Time * Ac	0.11 M	0.7*	0.076 M	2.289*	0.004	3.230*	0.937 L	1470.1*
	Error	0.52 L		0.10 M		0.004		0.001	
Citrate	Time	0.08 M	68.3*	0.56 L	85.43*	0.166 L	5.55**	0.024S	124.23*
	Cit	0.85 L	720.1*	0.281 L	42.81*	0.306 L	10.234*	0.109 M	553.60*
	Time * Cit	0.03S	8.18*	0.077 M	2.92*	0.152 L	1.275*	0.86 L	1096.3*
	Error	0.01S		0.082 M		0.374 L		0.002	
Chloride	Time	0.32 L	25.5*	0.559 L	201.44*	0.019S	73.091*	0.015S	239.72*
	Cl	0.24 L	18.7*	0.330 L	119.07*	0.96 L	3627.4*	0.216 L	3258.4*
	Time * Cl	0.30 L	5.9*	0.076 M	6.875*	0.008	7.875*	0.767 L	2889.4*
	Error	0.16 L		0.035S		0.003		0.001	
Nitrate	Time	0.21	22.9*	0.532	100.81*	0.011	30.300*	0.022	146.61*
	Nit	0.51	55.04*	0.339	64.27*	0.98	2652.1*	0.106	689.44*
	Time * Nit	0.10	2.928	0.063	3.002*	0.002	1.669*	0.868	1400.13*
	Error	0.11		0.066		0.004		0.002	

The significant effect of each factor on the parameters is achieved from the two-way ANOVA.

The positive sign (+) indicates positive correlations and the negative sign (−) indicates negative correlations while one star (*) indicates weak correlation value (< 0.7) and two stars (**) indicate strong correlation (≥ 0.7); no star indicates no correlation. L, M and S indicate the effect size.

## Discussion

A model non-pathogenic bacterial strain was locally isolated and genetically identified as *B. subtilis* OQ119616 and taken as the case-study organism in this work. Its capability and magnitude to perform BICCP (the *Bacillus* induced calcium carbonate precipitation), the governing metabolism of the process as well as the anticipated applications of the product were pursued. Growth of *B. subtilis* (O.D._660 nm_ and protein contents) continued for 5 days in minimal medium, supplemented with urea and one of successively increasing levels of different calcium salts: chloride “Cl”, acetate “Ac”, nitrate “N”, citrate “Ci”; concentrations given to the bacterium were dependent on the type of salt, which in turn is contingent on their specific solubility coefficients.

The highest growth and lowest (O.D. and protein) were shown at 15 g/L of calcium chloride and at 0.23 g/L of calcium citrate; respectively.

Supplemented with any of the calcium salt concentrations, *B. subtilis* exhibited a potent capability of BICCP. Quantitatively, the amounts of CaCO_3_ produced/precipitated exhibited the following order: chloride > nitrate > acetate > citrate. Achal and Pan^9^ recorded dependence of calcium carbonate amounts on calcium sources. Vice versa, the conversion efficiency percentage (CE%) of calcium salts given relative to the CaCO_3_ precipitated (weight of calcium salt given *100/ weight of calcium carbonate produced/precipitated) exhibited an attitude different from absolute quantities, as the highest percentage of conversion efficiency was induced by citrate i.e. citrate was the most efficient salt to be converted to calcium carbonate while calcium chloride exhibited the lowest efficiency, despite producing the highest quantities of calcium carbonate. Second to citrate is acetate CE%. The findings in this work indicate the influential participation of the anion in calcium salts i.e. acetate vs citrate, chloride or nitrate, i.e. the significance of the anions in BICCP quantities. Characterization of BICCP started by the description of their polymorphs, as calcification is strain-specific, due to differences in urease expression^8^ and correlated to the type of calcium salt provided^9^. In this work, scanning electron microscopy (SEM) displayed four different morphologies of BICCP, which are needle-like produced from calcium nitrate, spherical from calcium citrate, irregular from calcium acetate and rhombohedral for calcium chloride These morphs are valerite (needle) according to Chakrabarty and Mahapatra^26^ and Lee et al.^27^, aragonite (spherical), and calcite (rhombohedral) according to Barhoum et al.^28^.

BICCP was shown to contain micro-and nanoparticles resulting in compatibility with several applications.

The morphologies of calcium carbonate have been repeatedly reported to depend on the type of calcium salt provided, the growth conditions of e.g., pH or temperature as well as on type of the microorganism performing the MICP process, the latter acts as the template or nucleation site for calcium carbonate precipitation. In this respect. Ronholm et al.^29^ observed that mineralogical features of euhedral or anhedral precipitates are indicative of biogenic origin. Also, Hammes et al.^30^ clearly correlated the striking differences in morphology of large calcium carbonate crystal aggregates with different microbial species with attributes of *Bacillus sphaericus* group. Payandi-Rolland et al.^7^ recorded three main morphologies of crystals (exclusively calcite) associated with Oscillatoriaceae, Phormidiaceae and their EPS; rhombic sparite crystals are wrapped in EPS. Furthermore, Growth rate—different calcium salts may interact differently under thermal conditions, leading to morphologically distinct products. In addition, different calcium salts may interact differently, leading to morphologically distinct products^31^. The rapidly dissolving salts (like CaCl_2_), favoring fast nucleation and often leading to amorphous or less stable morphologies while slower-releasing salts (e.g. Calcium sulfate and Calcium oxalate) might promote controlled crystal growth, allowing more ordered structures like calcite to form^32^. Certain anions may adsorb onto the growing crystal surfaces, selectively inhibiting specific crystal faces, thus altering morphology e.g., elongation or flattening of crystals^30^. The pH also influences which polymorph (calcite, aragonite, vaterite) is thermodynamically or kinetically favored^33^. While temperature is not directly tied to calcium source, the interaction between calcium source and temperature can affect Polymorph selection e.g., aragonite is favored at higher temperatures^34^. Furthermore, some calcium salts may contain trace impurities (e.g., Mg^2+^, Fe^3+^) that can inhibit or stabilize specific polymorphs, such as Mg^2+^ stabilizing aragonite^33^.

The bacterial cells remained alive after 30 days in CaCO_3_-rich medium. Viability of bacterial cells has been considered for two purposes: tolerability of the cells and sustainability of the process. Viability inside media containing precipitates indicates the tolerability of bacterial cells to survive harsh conditions of CaCO_3_ and its accompanying alkalinity, on the one side. On the other hand, prolonged cells viability indicates possible durability of BICCP with its applicability for 30 days or may be for longer times than 30 days, the experimental period. Of particular importance of high tolerability and durability is the possible continuity of CO_2_ capture and sequestration, which is crucial to the environment and considered in this work (discussed later).

During growth of *B. subtilis*, the pH of the medium was modulated to the alkaline side, which is substantial to suit the process and stability of MICP. The elevated Δ pHs of the culture media to alkalinity (relative to the pH of control, which received none of the treatments i.e. neither calcium nor urea) exhibited dependence on the type of calcium salt and concentration given to *B. subtilis* in the following order: chloride > nitrate > acetate > citrate, which coincided with the amount of MICP formed. The highest growth magnitude coincided with the highest pH value and MICP. Alkalization is a prerequisite for MICP, as calcification occurs under alkaline pH values from 8.7 to 9.5^35,36^. When pH levels decrease, carbonates tend to dissolve rather than precipitate^37^. In this work, citrate induced the least elevation in Δ pHs while acetate induced the highest Δ pH shifts; nitrate and chloride were in between. The continuous ability of elevating pH of the culture media to high pH values, in turn, might have caused a slow and long-lasting lag phase in growth of *B. subtilis*, but a favorable condition for precipitating calcium carbonate.

The pH elevation is ascribed mainly to the accumulation of ammonia, which in turn could be ascribed to the activity of the urease enzyme. Urease activity is recognized as the main driver of ammonia accumulation^38–40^ and Ca^2+^ has been reported as inductive to a sharp enhancement of urease activity resulting in a pronounced upregulation, with differences among strains^8^. Urease activity and ammonia content demonstrated a conclusive difference between acetate and citrate, the highest and lowest activity was accompanied with the highest and lowest BICCP, respectively. The discrepancy between both calcium salts of citrate and acetate confirms the role of urease in MICP production, via their product ammonia-induced pH elevation, which has been assigned in the literature, applies also in the case-studied strain (*B. subtilis* OQ119616). This concept also applies to calcium chloride and calcium nitrate as their highest activities were accompanied by the highest amounts of MICP and vice versa. Hammes et al.^30^ recorded an increase in urease activity of certain *Bacillus sphaericus* isolates up to tenfold in the presence of 30 mM calcium compared with the absence of Ca^2+^. Urease gene diversity, substrate affinities (Km) and maximum hydrolysis rates (Vmax) of crude enzyme extracts revealed significant differences among the various isolates related to *Bacillus sphaericus* group. Urease activity is assessed by its final product `´ammonia``. In this work, urease exhibited a one shot activity for the first 24 h of culturing only while ammonia contents sustained its levels throughout the experimental period.

Calcium salts are composed of calcium cations (Ca^2+^) and various counter-ions (e.g., Cl^−^ in CaCl_2_, NO_3_^−^ in Ca(NO_3_)_2_, CH_3_COO^−^ in Ca(CH_3_COO)_2_). These anions can affect urease activity through Direct interaction with the enzyme, potentially altering its conformation, Changes in water structure and enzyme hydration, based on the Hofmeister series, which ranks ions by their ability to stabilize or destabilize proteins and /or Competitive binding or inhibition—some anions may interfere with essential cofactors or active sites on the enzyme^41^. Furthermore, different calcium salts can shift the pH of the surrounding environment upon dissolution. Urease has an optimal activity range at neutral to slightly alkaline pH (around 7.5–8.5). Some salts may lower or raise the pH, indirectly affecting enzyme activity^42^. For instance, Calcium acetate may raise pH slightly while Calcium nitrate or calcium chloride may cause acidification. Even small pH deviations can lead to significant changes in enzyme structure and performance. Salts contribute to the ionic strength of the solution, which in turn can affect the electrostatic environment around the enzyme^43^. The electrostatic environment cause protein aggregation or unfolding at high concentrations and Influence enzyme–substrate interactions, potentially enhancing or reducing catalytic efficiency. Gene expression related to urease synthesis is induced by calcium. Urease is a metalloenzyme that typically requires nickel ions (Ni^2+^) for activity. High concentrations of Ca^2+^ from different calcium sources might Displace or compete with Ni^2+^ or other essential metal ions. Alter enzyme structure or prevent proper folding, leading to reduced activity. Urease that is produced by microbes is susceptible to calcium sources as it can influence Cell membrane integrity and ion transport, affecting enzyme production and secretion. Metabolic activity determines the amount and activity level of urease.

In addition to urease, nitrogenase activity of *B. subtilis* cultures is another ammonia producing enzyme exists in *B. subtilis*. As the activities of these enzymes are subjected to the effects of the imposed treatments, they have been followed throughout the life span of *B. subtilis* at the experimental treatments of calcium salts and concentrations.

Upon precipitating calcium and carbon dioxide to form CaCO_3_ via MICP, the cycle of water, land, and atmosphere is completely connected. As calcium carbonate precipitation occurs according to the following formula: Ca^2+^  + CO_2_ + H_2_O → CaCO_3_, i.e. CO_2_ is concomitantly fixed into CaCO_3_ at equi-molar ratios. The latter i.e. carbon precipitation during BICCP is targeted in this study to be utilized within the scope of CO_2_ removal (CDR) from the atmosphere in open or closed systems. The ``BICCP-based CDR`` is anticipated to be one among other underway research trials for air decarbonization emerging technologies such as CO_2_ sorption, electrochemical reduction or underground storage. For example, Li et al.^44^ introduced low-cost materials called ‘charged-sorbents’ that can rapidly capture carbon dioxide from ambient air by means of (bi)carbonate formation. Also, Nozaki et al.^45^ introduced a cost-effective method to produce valuable hydrocarbons from CO_2_ conversion e.g. via electrochemical reduction into fuel (ethylene and propane) and other chemicals on a basic silver (Ag) electrode. In addition, a new method for carbon storage that accelerates the formation of carbon dioxide hydrates using a chemical-free process has been developed. Bhati et al.^46^ stated that the latter technique converts gigatons of CO_2_ into stable ice-like materials for ocean burial, which could significantly reduce the atmospheric carbon levels and address climate change. Utilization of MICP would be regarded as exploitation of a naturally occurring biogeochemical phenomenon in technical purposes, which might be relevant for decarbonizing filters of exhausts of carbon dioxide producing facilities such as breweries, factories or closed systems, etc.; thus, it prevents CO_2_ from releasing into the atmosphere.

Based on the above assumption, the economic feasibility of exploiting the `BICCP-based CDR` has been postulated and calculated in three terms. First, is the Economic Feasibility 1 (EF1) of BICCP in CDR as moles CO_2_ fixed/kg salts, second is the Economic Feasibility 2 (EF2) of BICCP in CDR as moles CO_2_ fixed/ US$ and third is Economic Feasibility 3 (EF3) of MICP in CDR as US$c/m^3^ atmosphere purged from CO_2_. In this regard, acetate would be considered the most applicable salt in CO_2_ capture and sequestration, considering its conversion efficiency and cost efficiency (EF 1, EF 2 and EF 3), as acetate exhibited the least expensive salt in relation to the amount of CO_2_ fixed. Indeed, both conversion efficiency and cost efficiency (EF 1, EF 2 and EF3) were the highest at citrate, based on mathematics. However, citrate was ruled out from comparison and consideration owing to the fact of its scarce solubility, which renders its applicability inefficient conclusively. Therefore, combining solubility restrictions with conversion efficiency and economic feasibility points to acetate as the most applicable/ efficient salt for BICCP among the other salts and hence CO_2_ capture and sequestration. Acetate would be also applicable for its possible efficient recovery (if acidified by acetic acid) and reuse for endurance of the process.

Achieving the proper balance of pH of the environment, the type of calcium salt used, the concentration of both calcium ions and urea maximizes the conversion efficiency of calcium by a microorganism into calcium carbonate precipitation (MICP). Meanwhile, optimizing MICP conditions minimizes negative impacts like ammonia emissions and reduces substrate waste, which may contribute to achieving the twelfth UN Sustainable Development Goal concerning resource use when utilizing MICP for engineering applications. Spencer et al.^47^ reported Increased MICP efficiency via adding ammonium chloride. In this respect, Erdmann and Strieth^48^ reported that critically described the complexity and interactions of key influencing factors involved in ureolytic-based MICP. The urea and calcium concentrations affect the metabolism of ureolytic microorganisms, the efficiency of CaCO_3_ formation, and the morphology of the formed crystals^48–50^. The ideal efficiency of stoichiometric CaCO_3_ formation requires an equimolar amount of urea and calcium^49–51^, Optimization of these concentrations has been a central aspect of many studies dealing with MICP. Also, Qabany and Soga^52^ investigated the influence of different equimolar concentrations of urea and calcium in the range between 0.25 and 1 M on MICP. With increasing concentration of the calcination solution, the size of the CaCO_3_ crystals increased from 3–5 µm to up to 35 µm, while the uniformity of the distribution of the crystals and the permeability of the sample decreased. In addition, Cheng et al.^53^ found that the conversion efficiency of MICP remains approximately the same for pH values of 4–8.1, which has been rapidly decreasing at pH values below 4 to be 60% at pH 3.5, 10% at pH 3.0 and no CaCO_3_ precipitation at pH value of 2.5 and lower. In a similar study, Lai et al.^54^ also described a pH value which acts as a deactivation threshold for MICP. with Sporosarcina pasteurii DSM 33; below such a threshold that is dependent on the cell density, no CaCO_3_ precipitation occurs.

In addition to carbon capture and sequestration, the applicability of BICCP has been extended and checked in a number of possibilities. Namely, these applications are improving sand soil characteristics to be more cultivable, adsorption of polluting heavy metals from sewage effluents for purging and reuse and third healing concrete cracks. First, the permeability for water is a coherent chronic defect in sand soil, which extends in vast areas on earth. In turn, low water holding capacity limits agriculture and food production for the ever-increasing population on earth. In this work, field capacity (the maximum water holding capacity) was assessed to evaluate the alteration in soil characteristics concerning water strategy as influenced by *B. subtilis*–based MICP. The results obtained show that the field capacity of a sand soil column was enhanced, indicating that the MICP formed was accompanied with improved soil characteristics to hold more water and hence its agricultural capacity. The lower part of the sand column preserved more water than the upper part, due to gravitational force. As it is anticipated and targeted, the elevation of the filed capacity was stimulatory to germination of *V. faba* seeds and growth of their plumules. The highest germination percentage was recorded at BICCP-treated sand samples (supplemented with healing cocktail) as 80% relative to the total number of seeds tested while the only-watered samples exhibited only 20%. Plumule length was also longer in BICCP-treated sands. In this respect, MICP-improved soil characteristics have been described elsewhere. For instance, Ng et al.^55^ outlined an overview of the factors affecting microbial-induced calcite precipitation and its potential application in soil improvement. Also, Martinez et al.^56^ conducted experimental optimization of microbial induced carbonate precipitation for soil improvement. In addition, Chu et al.^57^ described the microbially induced calcium carbonate precipitation on surface or in the bulk of soil.

BICCP was also used to adsorb heavy metals from sewage effluents for partial purging and cleansing of contaminants in waste/sewage effluents for environment, hygiene and reuse. In this work, BICCP was accompanied with adsorption of some mineral elements (heavy metals); namely, Pd, Zn, Cd and Cu. The results revealed a sharp decrease in soluble heavy metal levels in the effluent, accompanying the formed calcium carbonate precipitations. It is noteworthy denoting that chemically added CaCO_3_ could not decrease mineral contents, confirming that microbial cells that adsorb the heavy metals on walls of *Bacillus* cells. In agreement with these findings, Achal et al.^58^ reported the remediation of copper-contaminated soil by *Kocuria flava* CR1 based on microbially induced calcite precipitation. Also, Li et al.^40^ and Kang et al.^59^ reported the bioremediation of Cd by microbially induced calcite precipitation. Ma et al.^60^ described competitive removal of water-borne copper, zinc and cadmium by a CaCO_3_-dominated red mud. Earlier, Pan^61^ considered MICP as a promising way to in situ immobilize heavy metals in groundwater and sediment. Furthermore, Yin and Zhu^62^ reported an In situ remediation of metal contaminated lake sediment using naturally occurring, calcium-rich clay mineral-based low-cost amendment. Therefore, the results in this work conclude that the herein studied organism *B. subtilis* and its BICCP could lead to a positive role in validating drainage effluents for reuse, especially in water-poor countries.

Third, BICCP could repair artificial concrete cracks of 1–2 mm wide and 10 mm long in Petri dishes; progression of healing was taking place throughout 7 days. In this respect, the healing of cracks in concrete by MICP was repeatedly tested, and described as concrete remediation^63–65^, self-healing concrete^66–68^, cemented strength of sand^69,70^, Lee and Park^71^ followed current challenges and future directions for bacterial self-healing. Menon et al.^72^ screened fungi for their potential application of self-healing concrete. Feng and Montoya^73^ studied the Influence of confinement and cementation level on the behavior of microbial-induced calcite precipitated sands under monotonic drained loading. Castro-Alonso et al.^13^ described microbiological and molecular concepts of MICP and its potential in bioconcrete. Achal et al.^74^ reported the improved strength and durability of fly ash-amended concrete by microbial calcite precipitation. Lin et al.^75^ evaluated the mechanical behavior of sands treated by MICP. Montoya and De Jong^76^ described stress–strain behavior of sands cemented by microbially induced calcite precipitation.

In this study, a two-way analysis of variance (ANOVA) was employed to evaluate the influence of different salt types and incubation times on calcium carbonate precipitation (CaCO₃) by *B.* subtilis.

The results revealed that both the type of salt and the incubation time had significant main effects on calcium carbonate precipitation (*p* < 0.05). This suggests that ion composition in the medium and the duration of bacterial activity both play critical roles in promoting biomineralization by *Bacillus* strains.

Importantly, the interaction effect between salt type and time was also statistically significant, indicating that the influence of salt on CaCO₃ precipitation is not consistent across all time points. This finding underscores the dynamic and condition-dependent nature of bacterial mineralization processes.

The enhanced precipitation in some salts could be attributed to their role in modulating bacterial urease activity.

The time-dependent increase in CaCO₃ precipitation aligns with the typical growth kinetics of *Bacillus* spp., where active metabolism, urease activity increase over time, facilitating the accumulation of carbonate ions and subsequent mineral nucleation.

These results are consistent with previous studies that report optimal biomineralization under specific ionic and temporal conditions^40,77^. The interaction between time and salt further highlights the importance of optimizing both environmental and growth conditions to maximize biomineralization yields.

## Electronic supplementary material

Below is the link to the electronic supplementary material.

Supplementary Material 1

## Data Availability

The data will be provided under request from the corresponding author.
