# Supplementary material for: Assessment of ecofriendly carbon capture using Bacillus subtilis induced calcium carbonate precipitation with focus on applications mechanisms and cost efficiency
Source: Sci Rep. 2025 Jul 1;15:21906. doi: 10.1038/s41598-025-06688-1 (PMC12215896; doi:10.1038/s41598-025-06688-1)
Supplement: Supplementary file 1 — Supplementary Material 1 [file 41598_2025_6688_MOESM1_ESM.docx]

**Assessment of ecofriendly carbon capture using *Bacillus subtilis* induced calcium carbonate precipitation with focus on applications mechanisms and cost efficiency**

Amal W. Danial, Raghad M.M. Hasan, Ghada Abd-Elmonsef Mahmoud and Refat Abdel-Basset

Botany and Microbiology Department, Faculty of Science, Assiut University, 71516 Assiut (Egypt)

Corresponding author: daniala.w@aun.edu.eg

**Table S1.** One-way ANOVA summary for the effect of different calcium salts (acetate, citrate, chloride and nitrate on [*Bacillus subtilis* growth (OD)]. A significant *F*-value (p < 0.05) indicates differences among treatment groups.

| **ANOVA** | | | | | | |
| --- | --- | --- | --- | --- | --- | --- |
|  | | Sum of Squares | df | Mean Square | F | Sig. |
| Control | Between Groups | .000 | 3 | .000 | .000 | 1.000 |
|  | Within Groups | .002 | 8 | .000 |  |  |
|  | Total | .002 | 11 |  |  |  |
| Ca-acetate | Between Groups | .602 | 3 | .201 | 1.507 | .285 |
|  | Within Groups | 1.064 | 8 | .133 |  |  |
|  | Total | 1.666 | 11 |  |  |  |
| Ca-citrate | Between Groups | 1.173 | 3 | .391 | 165.464 | .000 |
|  | Within Groups | .019 | 8 | .002 |  |  |
|  | Total | 1.192 | 11 |  |  |  |
| CaCl_2_ | Between Groups | 1.837 | 3 | .612 | 1679.909 | .000 |
|  | Within Groups | .003 | 8 | .000 |  |  |
|  | Total | 1.840 | 11 |  |  |  |
| Ca-nitrate | Between Groups | 1.252 | 3 | .417 | 22.712 | .000 |
|  | Within Groups | .147 | 8 | .018 |  |  |
|  | Total | 1.399 | 11 |  |  |  |

**Table S2.** One-way ANOVA summary for the effect of different calcium salts (acetate, citrate, chloride and nitrate on [*Bacillus subtilis* growth (protein)]. A significant *F*-value (p < 0.05) indicates differences among treatment groups.

| **ANOVA** | | | | | | |
| --- | --- | --- | --- | --- | --- | --- |
|  | | Sum of Squares | df | Mean Square | F | Sig. |
| Control | Between Groups | .000 | 3 | .000 | .000 | 1.000 |
|  | Within Groups | .031 | 8 | .004 |  |  |
|  | Total | .031 | 11 |  |  |  |
| Ca-acetate | Between Groups | .066 | 3 | .022 | 13.493 | .002 |
|  | Within Groups | .013 | 8 | .002 |  |  |
|  | Total | .079 | 11 |  |  |  |
| Ca-citrate | Between Groups | .073 | 3 | .024 | 16.724 | .001 |
|  | Within Groups | .012 | 8 | .001 |  |  |
|  | Total | .084 | 11 |  |  |  |
| CaCl_2_ | Between Groups | .191 | 3 | .064 | 56.283 | .000 |
|  | Within Groups | .009 | 8 | .001 |  |  |
|  | Total | .200 | 11 |  |  |  |
| Ca-nitrate | Between Groups | .046 | 3 | .015 | 12.707 | .002 |
|  | Within Groups | .010 | 8 | .001 |  |  |
|  | Total | .056 | 11 |  |  |  |

**Table S3.** One-way ANOVA summary for the effect of different calcium salts (acetate, citrate, chloride and nitrate on ammonia content by *Bacillus subtilis*. A significant *F*-value (p < 0.05) indicates differences among treatment groups.

| **ANOVA** | | | | | | |
| --- | --- | --- | --- | --- | --- | --- |
|  | | Sum of Squares | df | Mean Square | F | Sig. |
| Control | Between Groups | .000 | 4 | .000 | .000 | 1.000 |
|  | Within Groups | .013 | 10 | .001 |  |  |
|  | Total | .013 | 14 |  |  |  |
| Ca-acetate | Between Groups | 6.133 | 3 | 2.044 | 421.044 | .000 |
|  | Within Groups | .039 | 8 | .005 |  |  |
|  | Total | 6.172 | 11 |  |  |  |
| Ca-citrate | Between Groups | .010 | 3 | .003 | 24.568 | .000 |
|  | Within Groups | .001 | 8 | .000 |  |  |
|  | Total | .011 | 11 |  |  |  |
| CaCl_2_ | Between Groups | .782 | 3 | .261 | 46.336 | .000 |
|  | Within Groups | .045 | 8 | .006 |  |  |
|  | Total | .828 | 11 |  |  |  |
| Ca-nitrate | Between Groups | 11.850 | 3 | 3.950 | 561.489 | .000 |
|  | Within Groups | .056 | 8 | .007 |  |  |
|  | Total | 11.906 | 11 |  |  |  |

**Table S4.** One-way ANOVA summary for the effect of different calcium salts (acetate, citrate, chloride and nitrate on urease activity by *Bacillus subtilis*. A significant *F*-value (p < 0.05) indicates differences among treatment groups.

| **ANOVA** | | | | | | |
| --- | --- | --- | --- | --- | --- | --- |
|  | | Sum of Squares | df | Mean Square | F | Sig. |
| Control | Between Groups | .000 | 3 | .000 | .000 | 1.000 |
|  | Within Groups | .010 | 8 | .001 |  |  |
|  | Total | .010 | 11 |  |  |  |
| Ca-acetate | Between Groups | 5.771 | 3 | 1.924 | 421.044 | .000 |
|  | Within Groups | .037 | 8 | .005 |  |  |
|  | Total | 5.807 | 11 |  |  |  |
| Ca-citrate | Between Groups | .009 | 3 | .003 | 24.568 | .000 |
|  | Within Groups | .001 | 8 | .000 |  |  |
|  | Total | .010 | 11 |  |  |  |
| CaCl_2_ | Between Groups | .736 | 3 | .245 | 46.336 | .000 |
|  | Within Groups | .042 | 8 | .005 |  |  |
|  | Total | .779 | 11 |  |  |  |
| Ca-nitrate | Between Groups | 11.149 | 3 | 3.716 | 561.489 | .000 |
|  | Within Groups | .053 | 8 | .007 |  |  |
|  | Total | 11.202 | 11 |  |  |  |

**Table S5.** One-way ANOVA summary for the effect of different calcium salts (acetate, citrate, chloride and nitrate on CaCO_3_ precipitation content by *Bacillus subtilis*. A significant *F*-value (p < 0.05) indicates differences among treatment groups.

| **ANOVA** | | | | | | |
| --- | --- | --- | --- | --- | --- | --- |
|  | | Sum of Squares | df | Mean Square | F | Sig. |
| Control | Between Groups | .000 | 3 | .000 | .000 | 1.000 |
|  | Within Groups | .001 | 8 | .000 |  |  |
|  | Total | .001 | 11 |  |  |  |
| Ca-acetate | Between Groups | .310 | 3 | .103 | 117.564 | .000 |
|  | Within Groups | .007 | 8 | .001 |  |  |
|  | Total | .317 | 11 |  |  |  |
| Ca-citrate | Between Groups | .033 | 3 | .011 | 7.599 | .010 |
|  | Within Groups | .012 | 8 | .001 |  |  |
|  | Total | .045 | 11 |  |  |  |
| CaCl_2_ | Between Groups | 1.748 | 3 | .583 | 106.701 | .000 |
|  | Within Groups | .044 | 8 | .005 |  |  |
|  | Total | 1.792 | 11 |  |  |  |
| Ca-nitrate | Between Groups | .465 | 3 | .155 | 56.860 | .000 |
|  | Within Groups | .022 | 8 | .003 |  |  |
|  | Total | .487 | 11 |  |  |  |

**Table S6.** One-way ANOVA summary for the effect of different calcium salts (acetate, citrate, chloride and nitrate on nitrogenase activity by *Bacillus subtilis*. A significant *F*-value (p < 0.05) indicates differences among treatment groups.

| **ANOVA** | | | | | | |
| --- | --- | --- | --- | --- | --- | --- |
|  | | Sum of Squares | df | Mean Square | F | Sig. |
| Control | Between Groups | .000 | 3 | .000 | .000 | 1.000 |
|  | Within Groups | 5066.667 | 8 | 633.333 |  |  |
|  | Total | 5066.667 | 11 |  |  |  |
| Acetate | Between Groups | 27051.000 | 3 | 9017.000 | 40.693 | .000 |
|  | Within Groups | 1772.667 | 8 | 221.583 |  |  |
|  | Total | 28823.667 | 11 |  |  |  |
| Citrate | Between Groups | 107883.333 | 3 | 35961.111 | 392.303 | .000 |
|  | Within Groups | 733.333 | 8 | 91.667 |  |  |
|  | Total | 108616.667 | 11 |  |  |  |
| Chloride | Between Groups | 78922.917 | 3 | 26307.639 | 87.692 | .000 |
|  | Within Groups | 2400.000 | 8 | 300.000 |  |  |
|  | Total | 81322.917 | 11 |  |  |  |
| Nitrate | Between Groups | 73772.917 | 3 | 24590.972 | 231.444 | .000 |
|  | Within Groups | 850.000 | 8 | 106.250 |  |  |
|  | Total | 74622.917 | 11 |  |  |  |
